# Phosphorylation decelerates conformational dynamics in bacterial translation elongation factors

**DOI:** 10.1126/sciadv.aap9714

**Published:** 2018-03-14

**Authors:** Ariel Talavera, Jelle Hendrix, Wim Versées, Dukas Jurėnas, Katleen Van Nerom, Niels Vandenberk, Ranjan Kumar Singh, Albert Konijnenberg, Steven De Gieter, Daniel Castro-Roa, Anders Barth, Henri De Greve, Frank Sobott, Johan Hofkens, Nikolay Zenkin, Remy Loris, Abel Garcia-Pino

**Affiliations:** 1 Structural Biology Brussels, Department of Bio-engineering Sciences, Vrije Universiteit Brussel, Brussels, Belgium.; 2 Center for Structural Biology, VIB, Flanders, Belgium.; 3 Molecular Imaging and Photonics, University of Leuven, B-3001 Leuven, Belgium.; 4 Biomedical Research Institute, Hasselt University, B-3590 Hasselt, Belgium.; 5 Cellular and Molecular Microbiology, Department of Molecular Biology, Université Libre de Bruxelles, Brussels, Belgium.; 6 Biomolecular and Analytical Mass Spectrometry Group, Department of Chemistry, University of Antwerp, Antwerp, Belgium.; 7 Centre for Bacterial Cell Biology, Institute for Cell and Molecular Biosciences, Newcastle University, Baddiley-Clark Building, Richardson Road, Newcastle upon Tyne NE2 4AX, UK.; 8 Fluorescence Applications in Biology Laboratory, Department of Chemistry and Pharmacy, Ludwig-Maximilians-Universität München, Munich, Germany.; 9 School of Molecular and Cellular Biology, University of Leeds, Leeds LS2 9JT, UK.; 10 Astbury Centre for Structural Molecular Biology, University of Leeds, Leeds LS2 9JT, UK.; 11 Nano-Science Center, Department of Chemistry, University of Copenhagen, Universitetsparken 5, 2100 Copenhagen, Denmark.

## Abstract

Bacterial protein synthesis is intricately connected to metabolic rate. One of the ways in which bacteria respond to environmental stress is through posttranslational modifications of translation factors. Translation elongation factor Tu (EF-Tu) is methylated and phosphorylated in response to nutrient starvation upon entering stationary phase, and its phosphorylation is a crucial step in the pathway toward sporulation. We analyze how phosphorylation leads to inactivation of *Escherichia coli* EF-Tu. We provide structural and biophysical evidence that phosphorylation of EF-Tu at T382 acts as an efficient switch that turns off protein synthesis by decoupling nucleotide binding from the EF-Tu conformational cycle. Direct modifications of the EF-Tu switch I region or modifications in other regions stabilizing the β-hairpin state of switch I result in an effective allosteric trap that restricts the normal dynamics of EF-Tu and enables the evasion of the control exerted by nucleotides on G proteins. These results highlight stabilization of a phosphorylation-induced conformational trap as an essential mechanism for phosphoregulation of bacterial translation and metabolism. We propose that this mechanism may lead to the multisite phosphorylation state observed during dormancy and stationary phase.

## Teaser

Phosphorylation-induced conformational trap is an essential mechanism for phosphoregulation of bacterial metabolism.

## INTRODUCTION

Bacterial translation elongation is facilitated by three protein factors: elongation factors Tu (EF-Tu), Ts (EF-Ts), and G (EF-G). EF-Tu brings aminoacylated transfer RNAs (aa-tRNAs) to the ribosome. EF-Ts is a guanosine diphosphate (GDP)/guanosine triphosphate (GTP) nucleotide exchanger specific for EF-Tu, and EF-G is involved in the translocation of the translation ternary complex. These factors are under intense regulation as cells sense and adjust their metabolic function in response to variations in nutrient levels (*1*). EF-Tu, in particular, undergoes a number of posttranslational modifications, which strongly inhibit protein synthesis. These modifications include acetylation, methylation, and phosphorylation and can occur at several sites in the protein (*2*–*4*). It has been shown that these modifications play crucial roles in bacterial virulence, stress tolerance, dormancy, and sporogenesis (*5*–*8*).

Throughout the translation cycle, EF-Tu specifically binds aa-tRNAs, guanine nucleotides, EF-Ts, and Mg^2+^. It also hydrolyzes GTP, an activity that is significantly enhanced when bound to ribosomes. Besides its pivotal role in protein biosynthesis, recent works suggest that EF-Tu may also moonlight as a chaperone for diverse proteins (*9*–*11*). Not surprisingly, several known antibiotics target the activity of EF-Tu (*12*).

EF-Tu is a multidomain protein. The N-terminal domain (G domain) is a guanosine triphosphatase (GTPase) that is followed by two β-barrel domains, important for aa-tRNA binding (*13*–*15*). G-domain proteins generally function as molecular switches by alternating between inactive GDP-bound and active GTP-bound states (*16*). In the case of EF-Tu, the GTP-bound or closed state is characterized by a compact arrangement of all three domains that form a large interface where the acceptor stem and the aminoacylated CCA tail of tRNAs dock (*15*). In the GDP-bound or open state, its conformation is less compact and is characterized by a large cavity between the three domains. The conformational changes associated with hydrolysis of GTP to GDP have a profound effect on aa-tRNA binding and on the interaction of EF-Tu with the ribosome itself (*17*).

Bacterial EF-Tu is extensively modified during the different growth states by phosphorylation, methylation, and acetylation with hypermodifications occurring in the stationary phase (*5*, *18*–*20*). Phosphorylation of *Escherichia coli* EF-Tu at T382 was discovered more than 20 years ago (*2*), and since then, several other phosphorylation sites have been described (*18*). It has been suggested that phosphorylation at a single site is sufficient to inactivate EF-Tu and interfere with translation elongation (*2*, *21*). In *Bacillus subtilis*, phosphorylation of EF-Tu at T65 and T385 also inhibits translation and plays an important role in dormancy and sporogenesis (*8*). However, the mechanism by which phosphorylation inactivates EF-Tu remains unknown. Although one hypothesis states that phosphorylation of *E*. *coli* EF-Tu prevents it from forming ternary aa-tRNA–GTP–EF-Tu complexes (*22*), experiments on *B*. *subtilis* EF-Tu suggested that phosphorylation cripples its GTPase activity and prevents the release of GTP–EF-Tu complexes from the ribosome (*8*). Here, we characterize the activity and structure of EF-Tu phosphorylated at T382 (pEF-Tu_T382_), as well as mutants mimicking other in vivo prevalent phosphorylation states of EF-Tu. We show that modifications affecting the β-hairpin conformation of the switch I region of EF-Tu (that precludes the interaction of EF-Tu with aa-tRNAs) shift the conformational equilibrium of EF-Tu to the open state. This uncouples EF-Tu from nucleotide exchange–induced conformational dynamics and results in strong translation inhibition described in vivo and in vitro (*2*, *3*, *8*, *21*).

## RESULTS

### Doc phosphorylates EF-Tu at T382

We recently showed that the toxin Doc from the toxin-antitoxin module *doc/phd* hijacks the natural posttranslational modification cycle of EF-Tu to target translation elongation by phosphorylation of EF-Tu at T382 (*21*). In *E*. *coli*, this results in a strong protein synthesis inhibition and cell growth arrest (*21*, *23*). Therefore, to study the structural and functional effects of EF-Tu phosphorylation, we used Doc as a tool to produce pEF-Tu_T382_ in vitro. Mass spectrometry and Western blotting confirmed that EF-Tu was efficiently phosphorylated using this protocol (fig. S1, A and B).

### Interaction of EF-Tu and pEF-Tu_T382_ with nucleotides

**Fig. 1:**
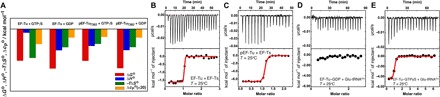
Thermodynamics of the interaction between EF-Tu and pEF-Tu_T382_ with nucleotides, EF-Ts and Glu-tRNA^Glu^. (**A**) Thermodynamic fingerprint of the interaction between EF-Tu and pEF-Tu_T382_ with nucleotides. The themodynamic parameters (Δ*G*, Δ*H*, −*T*Δ*S*, and Δ*c*_P_) that describe the interaction of EF-Tu with GDP and GTPγS are compared to those for the interaction of pEF-Tu_T382_ with GDP and GTPγS in the bar plot. ITC titration of EF-Ts into EF-Tu (**B**) and pEF-Tu_T382_ (**C**) at 25°C. ITC titration of pEF-Tu_T382_ into Glu-tRNA^Glu^ (**D**) and EF-Tu into Glu-tRNA^Glu^ (**E**) at 25°C.

Central to the function of EF-Tu in translation elongation is the interaction of its G domain with GTP and GDP (*17*). We used isothermal titration calorimetry (ITC) to characterize the effect of phosphorylation on the interaction of EF-Tu with di- and triphosphate guanine nucleotides [GDP and the nonhydrolyzable GTP analogs guanosine-5′-(γ-thio)-triphosphate (GTPγS) and 5′-guanylyl imidodiphosphate (GDPNP)]. At 20°C, the observed affinity of EF-Tu for GDP was 4.5 nM, whereas for GTPγS, it was 81 nM (Fig. 1A; fig. S2, A to H; and table S1). These results are consistent with the affinities measured with GDPNP (fig. S2, A to D, and table S1) and are in the range of earlier observations that show a preference of nucleotide-free EF-Tu for GDP (*24*) over GTP.

The observed affinity of pEF-Tu_T382_ for GDP was 5.7 nM, which is on the same order of magnitude as that measured for the nonphosphorylated protein (Fig. 1A; fig. S2, J and K; and table S1). Phosphorylation did not have a major effect on the affinity for GDPNP or GTPγS, with pEF-Tu_T382_ binding GTPγS with a *K*_d_ (dissociation constant) of 90 nM (Fig. 1A; fig. S2, M to O; and table S1). These results were confirmed by stopped-flow kinetics of nucleotides binding to EF-Tu and pEF-Tu_T382_, using the fluorescent compounds 2′/3′ *N*-methylanthraniloyl (MANT)–GDP and MANT-GTPγS. Phosphorylation at T382 did not affect the kinetics of GDP or GTPγS binding (fig. S3, A to F, and table S2) to either EF-Tu or pEF-Tu_T382_.

However, the thermodynamic binding profiles suggest that phosphorylation may have an impact on the structure and overall conformational landscape of the protein. In the nonmodified EF-Tu, the thermodynamic parameters of binding to GTPγS compared to GDP are quite contrasting (table S1). Binding to GTPγS is less enthalpically driven (Δ*H*) and is accompanied by a more severe entropic (Δ*S*) penalty and larger change in heat capacity (Δ*c*_P_). In addition, binding to GTPγS involves more residues in the contact interface as estimated from the measured Δ*H* and Δ*c*_P_ values (Fig. 1A, fig. S2P, and table S1). All these observations are consistent with structural observations, indicating that EF-Tu undergoes a large conformational rearrangement upon binding GTPγS. These changes involve burying hydrophobic residues and domain movements (around 980 Å^2^ of the surface become buried upon EF-Tu binding to triphosphate nucleotides) that lead to a closed and more compacted state compared to the GDP-bound open state, in which the three domains are more loosely arranged.

Notably, the thermodynamic binding pattern of GTPγS is significantly changed upon phosphorylation. The interaction between pEF-Tu_T382_ and GTPγS is more enthalpically driven; consequently, the binding entropic penalty is less severe and involves a lower Δ*c*_P_ (Fig. 1A and table S1). Thus, this binding pattern resembles more closely the interaction of EF-Tu with GDP, with fewer residues contributing to the binding interface than in the case of the nonphosphorylated protein (Fig. 1A and table S1). By contrast, the binding parameters for the interaction of pEF-Tu_T382_ with GDP remain largely unchanged compared to that of EF-Tu with GDP. Together, ITC and stopped-flow experiments suggest different modes of binding of GTPγS to phosphorylated and nonphosphorylated EF-Tu (Fig. 1A and table S1).

### Phosphorylation of EF-Tu impairs ternary complex formation

Next, we examined the effects of phosphorylation of EF-Tu at T382 on its interaction with elongation factor EF-Ts. EF-Ts catalyzes GDP to GTP exchange on EF-Tu (crucial for the formation of the ternary complex EF-Tu–GTP–aa-tRNA). EF-Ts has high affinity for EF-Tu in the subnanomolar range as measured by spectroscopic methods (*24*). We confirmed by ITC that EF-Tu binds EF-Ts with an affinity of 1.2 nM (Fig. 1B, fig. S2Q, and table S1). After phosphorylation, pEF-Tu_T382_ also bound EF-Ts with subnanomolar affinity (0.81 nM). Thus, the interaction of EF-Tu with EF-Ts seems largely unaffected by phosphorylation at Thr^382^. This is not surprising given that the site of phosphorylation at the second β-barrel domain (T382) is not involved in the EF-Ts–EF-Tu interface (Fig. 1C and figs. S2R and 4A) (*25*).

After guanine nucleotide exchange by EF-Ts, the EF-Tu–GTP complex attains the proper conformation suitable for recognizing and binding aa-tRNAs to form the ternary complex (EF-Tu–GTP–aa-tRNA) of translation elongation (*16*). It is in the form of these ternary complexes that EF-Tu delivers GTP and aa-tRNA to the ribosomal A site during translation. ITC titrations show that binding of Glu-tRNA^Glu^ to pEF-Tu_T382_ in the presence of GTPγS is strongly decreased compared to nonphosphorylated EF-Tu (Fig. 1, D and E, and fig. S2, S and T). This resembles the drop in affinity of Glu-tRNA^Glu^ for EF-Tu in the presence of GDP (fig. S2U). This is consistent with the decrease of in vitro ternary complex formation with Glu-tRNA^Glu^ in the presence of Doc (that phosphorylates EF-Tu in situ) or GDP compared to nonphosphorylated EF-Tu (fig. S4B). These results, together with the aforementioned nucleotide binding experiments, suggest that phosphorylation does not have a direct effect on the way pEF-Tu_T382_ interacts with nucleotides or EF-Ts, but might have a strong effect on the conformation and dynamic state of the enzyme, leading to a decreased affinity for aa-tRNAs.

### Structure of phosphorylated pEF-Tu_T382_

**Fig. 2:**
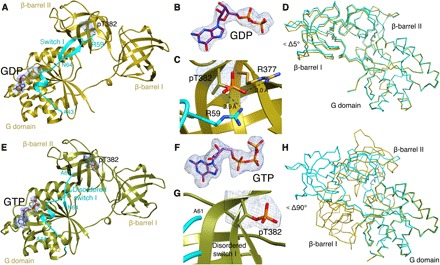
Effects of phosphorylation on the conformational state of pEF-Tu_T382_. (**A**) X-ray structure of the pEF-Tu_T382_–GDP complex. All three domains constituting EF-Tu (G domain and β-barrel I and II domains) are highlighted. The switch I region in the β-hairpin conformation is colored in cyan. The 2m*F*_o_-*DF*_c_ simulated-annealing omit map (σ = 1) corresponding to pT382 and GDP is shown as a blue mesh. The maps were calculated after removing pT382 and GDP from the model. (**B**) Close-up view of the electron density map presented in (A). (**C**) Details of the interactions of pT382 with R59 from the switch I β hairpin and R377 from the β-barrel II domain. (**D**) Cα representation of the phosphorylated (in cyan) and nonphosphorylated (in ocher) forms of EF-Tu bound to GDP. The two structures have been superimposed by aligning the G domain. (**E**) X-ray structure of the pEF-Tu_T382_–GTP complex. All three domains constituting EF-Tu (G domain and β-barrel I and II domains) are shown as in (A). The disordered switch I region is highlighted. The 2m*F*_o_-*DF*_c_ simulated-annealing omit map (σ = 1) corresponding to pT382 and GTP is shown as a blue mesh. The maps were calculated after removing pT382 and GTP from the model. (**F**) Close-up view of the electron density map presented in (A) for GTP and pT382 (**G**). The lack of contacts between pT382 and the switch I region due to local disordered is also highlighted. (**H**) Cα representation of the phosphorylated (in cyan) and nonphosphorylated (in ocher) forms of EF-Tu bound to GTP. The two structures have been superimposed aligning the G domain.

EF-Tu variants from different bacteria have been crystallized in different conformations and in complex with a myriad of partners. In these studies, two main types of conformations are observed: a closed one associated with GTP binding and an open one associated with GDP binding (*13*, *26*). However, there is little information regarding the effects of posttranslational modifications on the structure and molecular mechanism of the protein. The structure of the pEF-Tu_T382_–GDP complex determined at 2.8 Å shows an open conformation similar to that observed for EF-Tu in the GDP-bound state (Fig. 2, A to C, and table S3). The binding of GDP seems unaffected by the phosphorylation of T382 (Fig. 2, A and B), suggesting that long-range effects do not affect the GDP binding site on the G domain. The nucleotide adopts the same conformation observed in the crystal structure of the nonphosphorylated EF-Tu–GDP complex (*26*). The major structural difference is a 5° rotation of the β-barrel I domain relative to the G domain and β-barrel II domain as well as a local perturbation at the N-terminal side of the switch II region of the G domain (Fig. 2D and fig. S5A). In addition, β-barrel II is slightly closer to the G domain in the phosphorylated protein, with R59 from the switch I region directly contacting the phosphate group of pT382 (Fig. 2C).

To assess the effects of phosphorylation on the closed state of EF-Tu (the state compatible with aa-tRNA binding), we determined the crystal structure of the pEF-Tu_T382_–GTP complex at 2.8 Å resolution by MR-SAD (phasing using molecular replacement combined with single anomalous dispersion). The structure shows that, upon phosphorylation, even after GTP binding, the protein adopts an open state similar to that observed for the GDP-bound pEF-Tu_T382_ (Fig. 2, E to H). Consequently, in the pEF-Tu_T382_–GTP complex, the β-barrel II domain is rotated ~90° around its barrel axis compared to the structure of nonphosphorylated EF-Tu–GTP (Fig. 2H). When bound to GTP, the switch I region of pEF-Tu_T382_ is largely disordered (Fig. 2, E and G), specially the residues comprising the β hairpin. In the structure of the EF-Tu–GDP complex, this region involves several residues (including F46, I49, and D50) that make contacts and stabilized GDP in the open form. Moreover, in the EF-Tu–GTP complex, this region is directly involved in accommodating the γ-phosphate of GTP. The lack of electron density in the pEF-Tu_T382_–GTP structure suggests that the presence of the extra negative charge perturbs the local secondary structure (Fig. 2, E and H). This is likely due to the combined effect of the additional phosphate group on the nucleotide and the negative charge on pT382 that precludes the phosphorylated protein from collapsing into a more compact structure. It also suggests that anchoring the switch I β hairpin to the phosphorylated T382 (as observed in the pEF-Tu_T382_–GDP complex) is not the only requisite for trapping the protein in an open state. In addition, because the GTP binding site is altered in the open conformation, the γ-phosphate of GTP is not properly aligned in the active site and flips out toward the bulk solvent. This conformation is somewhat reminiscent of the intermediate GTP-bound states observed in the open EF-Tu during the nucleotide exchange reaction catalyzed by EF-Ts (fig. S5B). These intermediate states can accommodate triphosphate nucleotides in the presence or absence of Mg^2+^ without triggering the transition to a compact form (*27*). This dynamic behavior of the GTP-bound G domain has also been described for the equilibrium between active and inactive forms of GTP-ras_p21 complexes (*28*).

**Fig. 3:**
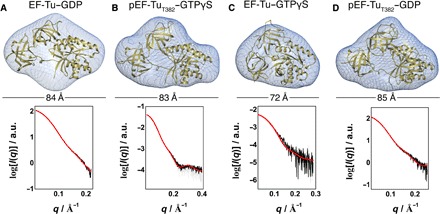
SAXS-based structural models of EF-Tu and pEF-Tu_T382_ in complex with GDP and GTPγS. Solution structures of pEF-Tu_T382_–GDP (**A**), pEF-Tu_T382_–GTPγS (**B**), EF-Tu–GDP (**C**), and EF-Tu–GTPγS (**D**). The model of *E*. *coli* EF-Tu–GTPγS in the closed state was reconstructed on the basis of the coordinate 1EXM (*66*) and is in very good agreement with the experimental SAXS data. The atomic model of the different complexes is superimposed on the ab initio calculated SAXS envelope shown as a blue meshed surface. In each case, the particle dimensions and experimental (in black) and model-derived (in red) SAXS curves are shown below each model. The calculated ab initio envelopes and experimental SAXS data of the complexes of pEF-Tu_T382_ with GDP, GTPγS, and GDPNP (table S4) are in good agreement with the crystal structures and strongly indicate that, upon phosphorylation, pEF-Tu_T382_ is in an open conformation independent of the bound nucleotide. a.u., arbitrary units.

We used small-angle x-ray scattering (SAXS) to probe the conformational state of EF-Tu and pEF-Tu_T382_ in solution, in the presence of di- and triphosphate nucleotides. The scattering data of EF-Tu bound to GDP confirmed that the protein adopts the typical open conformation observed in the crystal structure (Fig. 3A) (*26*). The solution scattering profile of pEF-Tu_T382_ bound to GDP also agrees with the open conformational state observed in the crystal structure (Figs. 2A and 3B). As expected, in the presence of GDPNP or GTPγS, nonphosphorylated EF-Tu is in the more compact, closed state (Fig. 3C and table S4) (*14*). As observed in the crystal structure (Fig. 2E) of pEF-Tu_T382_ in complex with GTP, in solution the complexes of pEF-Tu_T382_ with GTPγS or GDPNP show maximum dimensions (*D*_max_) that are ~10 Å bigger than the nonphosphorylated protein bound to GTPγS (Fig. 3, C and D, and table S4).

These results, together with the different crystal structures of pEF-Tu_T382_, suggest that pEF-Tu_T382_ predominantly populates predominantly an open state independent of the bound nucleotide. When looking in detail at the structure of the EF-Tu–GTPγS complex, T382 is buried in a hydrophobic interface created by the switch II region of the G domain and β-barrel II domain in the closed EF-Tu conformation (fig. S5, C and D). When we superimpose β-barrel II from pEF-Tu_T382_ onto the structure of the nonphosphorylated EF-Tu–GTPγS complex, it is clear that phosphorylation of T382 is incompatible with this closed conformation. Not only would the presence of a phosphate group drastically disturb the conformation of the switch II region (fig. S5D), but also the energetic penalty of burying the phosphate in a hydrophobic cavity would simply preclude the existence of such a conformation.

### Phosphorylation at T382 impairs EF-Tu dynamics

On the basis of the aforementioned observations, we hypothesized that at the structural level, phosphorylation interferes with the dynamic coupling between nucleotide binding and the conformational switch, intrinsic to EF-Tu. To provide insights into the conformational dynamics of EF-Tu, we used the single-pair Förster resonance energy transfer (spFRET) method.

**Fig. 4:**
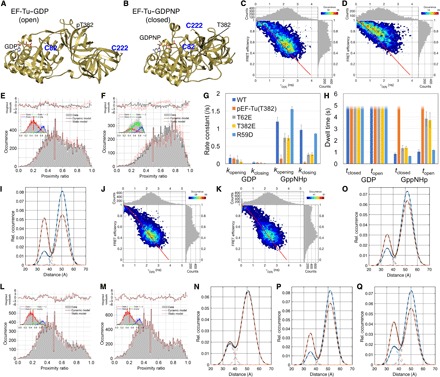
EF-Tu conformational dynamics in the presence of nucleotides assessed by spFRET. (**A** and **B**) Structural models of EF-Tu in the (A) open and (B) closed conformation. Dye attachment sites are Cys^82^ and Cys^222^. (**C** and **D**) Two-dimensional (2D) histograms of the FRET efficiency *E* versus the donor fluorescence lifetime τ_D(A)_ with 1D projections obtained after spFRET analysis of (C) EF-Tu–GDP and (D) EF-Tu–GDPNP. A 15% offset in *z* was used to reduce noise in the 2D histograms. Overlayed is (solid line) the theoretical relation between *E* and τ_D(A)_ in the absence of 1- to 10-ms FRET dynamics calculated using a τ_D_ estimated from the D-only population, an *R*_0_ = 53 Å, and Gaussian distributed fast dye fluctuations over 6 Å. (**E** and **F**) Static versus dynamic PDA analysis of EF-Tu in the presence of (E) GDP and (F) GDPNP. The insets illustrate the substrates used for dynamic PDA analysis [that is, the relative abundance of molecules that coincidently were only in the open (red) or closed (blue) state, and molecules that interconverted from one state to the other during diffusion through the probe volume (green)]. The (relative) area under the green substrate is directly proportional to the chance of observing a molecule that interconverted while diffusing through the probe volume; the larger this area, the more robustly can kinetic rate constants be derived from the data by dynamic PDA. (**G**) Bar chart of the *k*_opening_ and *k*_closing_ rate constants. The postfitting 95% relative confidence intervals were very low (<5%) for all parameters, except the *k* values (smaller than 20% for *k* > 0.25 s^−1^). The resulting rate constants are presented as the average ± SD of at least three independent measurements originating from at least two protein purification batches. The significances also apply to the data in (H). (**H**) Bar chart of the corresponding closed and open state dwell times. Dwell times > 5 ms were not quantified because of the high uncertainty of the corresponding interconversion rate constants (suggesting much slower conformational dynamics). For wild-type EF-Tu, the dwell time in the closed state was only slightly lower (*P* < 0.05) than that in the open state. GDPNP thus accelerates the rate of opening/closing with respect to GDP, and this frequent sampling of the closed state likely allows the protein to crystallize in this conformation. (**I**) Equilibrium distance distribution for different variants of EF-Tu, calculated from the *k*_opening_ and *k*_closing_ rate constants. (**J** and **K**) 2D histograms of the FRET efficiency *E* versus the donor fluorescence lifetime τ_D(A)_ with 1D projections obtained after spFRET analysis of (J) pEF-Tu_T382_–GDP and (K) EF-Tu–GDPNP. (**L** and **M**) Static versus dynamic PDA analysis [as in (E)] of (L) pEF-Tu_T382_ with GDP or (M) GDPNP. (**N** to **Q**) Equilibrium distance distribution for (N) pEF-Tu_T382_ and the different EF-Tu mutants (O) EF-Tu_T382E_, (P) EF-Tu_T61E_, and EF-Tu_R59D_ (Q).

spFRET probes 1- to 10-nm distances between fluorescent donor and acceptor attached to specific residues of a single freely diffusing protein. Distance fluctuations, equilibrium distribution, and kinetics of conformer interconversion on the 0- to 10-ms time scale, arising from conformational dynamics, can be quantified with spFRET. On the basis of accessible volume calculations, we selected possible solvent-exposed residues on EF-Tu for covalently attaching dyes. For residues C82 in the G domain and S222 in the β-barrel domain, the FRET-averaged interdye distance 〈*R*_DA_〉_*E*_ is predicted to be ~62 Å in the GDP state and ~41 Å in the GTP state (Fig. 4, A and B). These positions are sufficiently far from the phosphorylation site (Fig. 4, A to B), and mutations at S222C have negligible effects on nucleotide binding and the conformational cycle of EF-Tu (*29*, *30*). Direct titrations of nucleotides to EF-Tu_S222C_ showed that the mutant retains wild type–like affinity. Thus, we coupled the maleimide derivatives of ATTO 488 and Alexa Fluor 647 dyes to the thiol group of C82 and S222C (see fig. 6, A to G, for additional details on the labeling and analysis process) for the spFRET measurements.

The efficiency of the FRET process (*E*) and the average time the donor probe spent in the excited state (τ_D(A)_) were calculated for individual EF-Tu molecules (~100 pM) in the presence of 1 mM GDP (Fig. 4C and fig. S7A) or 1 mM GDPNP (Fig. 4D and fig. S7B). To quantitatively describe the conformational dynamics underlying the FRET histograms using photon distribution analysis (PDA), we set up a methodology that allowed global analysis of the GDP and GDPNP data sets (fig. S6B). The methodology involved the improved fitting robustness by globally linking fitting parameters, thereby reducing their number (fig. S6, C and D) and performing a detailed in silico analysis to accurately obtain the range of the rate constants (fig. S6, E to G). From this analysis, the interaction of EF-Tu with GDP could be adequately described using a model that assumed two invariable states with EF-Tu (on the <10-ms observation time scale) in a predominantly open conformation (Fig. 4E and fig. S7A). By contrast, in the case of interaction with GDPNP, we observed a nonlinear relation between *E* and τ_D(A)_ (Fig. 4D and fig. S7B), suggesting the presence of conformational dynamics on a 1- to 10-ms time scale. Accordingly, a model that assumed dynamically interconverting states described the interaction with GDPNP in a better way (Fig. 4F). The results show that GDPNP drastically increased the rate at which the protein opened and closed (Fig. 4, G and H, and tables S5 to S7), and that the open/closed equilibrium in EF-Tu-GDPNP is tipped toward the closed state (Fig. 4, F and I). These results are in agreement with crystallographic and SAXS data of EF-Tu with GDP and GDPNP and validate our system to monitor the EF-Tu conformational cycle (*14*, *26*).

We next performed a similar analysis of the interaction of phosphorylated EF-Tu with nucleotides (Fig. 4, J to N). In the presence of GDP, pEF-Tu_T382_ was observed predominantly in the open conformation, as was the nonphosphorylated protein (Fig. 4N and fig. S7C), and exhibited no conformational dynamics on the FRET time scale (Fig. 4, G, H, and L). In the presence of GDPNP, pEF-Tu_T382_ was also observed predominantly in the open (Fig. 4N and fig. S7D) and rather static conformation, switching only very slowly back and forth between open and closed states (Fig. 4, G, H, and M). These results indicate that phosphorylation decelerates conformational dynamics and tips the conformational equilibrium of pEF-Tu_T382_ in favor of the open conformation, thus decoupling nucleotide binding from the conformational cycle that EF-Tu requires for its proper function during translation elongation (that is, nucleotides no longer affect the conformation of the protein).

### T382E mimics the effect of phosphorylation on EF-Tu dynamics

The structure of pEF-Tu_T382_ suggests that the introduction of a negative charge in the loop containing T382 might prevent the compact state of EF-Tu associated with binding to GTP, thus trapping the protein in an open conformation. To challenge this hypothesis, we introduced the T382E mutation, a potential mimic of a phosphorylated Thr residue, and used spFRET to monitor the EF-Tu_T382E_ nucleotide-dependent conformational cycle. In the presence of GDP or GDPNP, EF-Tu_T382E_ remained preferentially in the open state similarly to pEF-Tu_T382_ (Fig. 4O and fig. S8, A and B). Detailed kinetic analyses additionally revealed that the opening/closing equilibrium was significantly decelerated, although it was still faster than the phosphorylated protein (Fig. 4, G and H, and fig. S9, A and B).

Thus, the presence of a negative charge in this position is sufficient to mimic the effects of phosphorylation in EF-Tu. The structure of the EF-Tu_T382E_–GDP complex is almost identical to that of pEF-Tu_T382_ bound to GDP or GTP (fig. S10A and Fig. 2, A and E). E382 that replaces the pT382 is involved in electrostatic interactions with the switch I region via R59, as seen in the case of pEF-Tu_T382_. All these results strongly suggest that EF-Tu_T382E_ is a bona fide structural and functional mimic of pEF-Tu_T382_.

### T61 is an allosteric hotspot involved in EF-Tu conformational dynamics

The conformation of the switch I region is intricately related with the open/closed state of G proteins and, in the case of EF-Tu, with the overall structural arrangement. On the basis of our structural observations on the link between the switch I region and phosphorylation of T382 at the β-barrel II domain, it is tempting to predict that posttranslational changes in EF-Tu, perturbing the switch I region β-hairpin conformation, would be an effective way of trapping the protein in an open conformational state.

T61 from the switch I region is another major phosphorylation site observed across bacterial proteomes. Therefore, we hypothesized that during translational arrest caused by T61 phosphorylation (*8*), the underlying molecular mechanism would again be decoupling of nucleotide binding from the EF-Tu conformational dynamics and trapping EF-Tu in an open conformational state.

To test this, we produced the T61E mutant to mimic *E*. *coli* EF-Tu phosphorylated at T61 (EF-Tu_T61E_). The structure of EF-Tu_T61E_ bound to GDP was very similar to that of EF-Tu_T382E_ and pEF-Tu_T382_ bound to GDP or GTP (fig. S10, A and B, and Fig. 2, A and E). E61 is located at the C-terminal β strand of the hairpin of the switch I region and interacts with K56 from the first β strand of this hairpin and thus stabilizes the overall β-hairpin conformation of the switch I region (fig. S10B). We also measured the influence of the T61E mutation on the nucleotide binding–dependent conformational switch using spFRET. In the presence of GDP, EF-Tu_T61E_ stays in the open form observed in the crystal structure (Fig. 4P and figs. 8C and 9C). As expected, in the presence of GDPNP, EF-Tu_T61E_ also slightly favored the open conformation (Fig. 4P and figs. 8D and 9D). In addition, the phosphomimic EF-Tu_T61E_ was significantly less dynamic than the wild-type protein (Fig. 4, G and H).

### Implications of the conformational trap on the structure-function interplay of EF-Tu

EF-Tu can be extensively modified posttranslationally (*31*). These modifications have a strong impact on bacterial metabolism and play an important role in shaping the phenotype of bacterial populations. Several of these modifications occur in response to nutrient starvation, stationary phase growth, or dormancy. From the 100 or more phosphopeptides observed in the *E*. *coli* and *B*. *subtilis* phosphoproteome, 10 are derived from EF-Tu (*2*, *18*, *19*). The phosphorylation sites are spread in all three domains of EF-Tu (table S8).

**Fig. 5:**
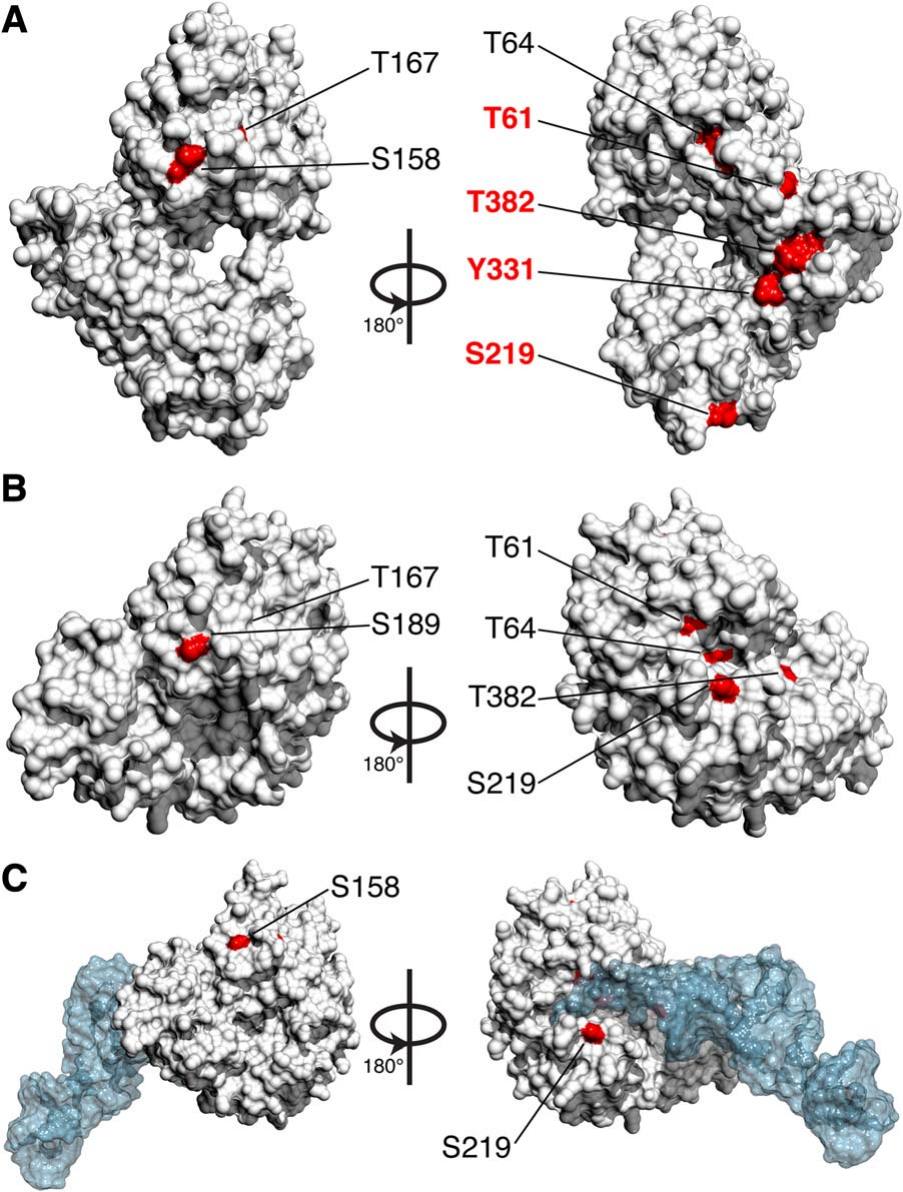
EF-Tu phosphorylation is intricately related with the protein conformational cycle. (**A**) In the closed state (EF-Tu–GTP complex), all the phosphorylation sites experimentally validated for EF-Tu are either buried or poorly accessible to the solvent. (**B**) In the closed conformation when bound to aa-tRNA (which is likely the more abundant state of the EF-Tu–GTP complex given the high affinity for aa-tRNA), only two phosphorylation sites are not involved in interactions with the aa-tRNA, and they have very low solvent accessibility. (**C**) In the open state, four sites become accessible by >25% and three others are partially accessible. This suggests that a modification trapping EF-Tu in an open state would increase the likelihood of additional modifications.

When we mapped these sites on the surface of EF-Tu in the closed conformation (Fig. 5A), the majority of the sites were fully buried, only partially exposed (less than 10%), or involved in aa-tRNA interactions, with only one site (S158) fully exposed (Fig. 5B). This implies that during normal translation, the closed-form EF-Tu would be rather protected from hyperphosphorylation. This is in stark contrast with the open conformation where at least five sites become exposed as a result of the nucleotide exchange (Fig. 5C). Hyperphosphorylation of EF-Tu has been described in several metabolic states. Considering that reversible protein phosphorylation is a true signal transduction device and that some of these sites are highly conserved (fig. S11), it is tempting to hypothesize that differential phosphorylation can set a threshold for a particular differentiated phenotypic state. Further experimental evidence will be needed to validate such a hypothesis.

## DISCUSSION

Translation factors have been observed in the phosphoproteomes of diverse organisms, suggesting that they are subject to regulation by phosphorylation (*7*, *18*, *19*, *32*, *33*). EF-Tu is extensively modified posttranslationally depending on the cellular state and growth conditions (*31*). Several of these modifications are directly involved in bacterial survival strategies and stress tolerance. In *E*. *coli*, the toxin Doc stalls translation and arrests growth by phosphorylating EF-Tu at T382 (*21*, *23*). In *Salmonella*, Doc is likely a key player in the differentiation of persister cells (*34*). Hyperphosphorylation of EF-Tu in *E*. *coli*, *Mycobacterium tuberculosis*, and *Mycobacterium smegmatis* reduces protein synthesis and cell growth (*20*). During nutrient starvation, *B*. *subtilis* forms a metabolically quiescent, highly resistant spore (*8*). This process is triggered by the phosphorylation of EF-Tu by YabT (at T65 and T385, the equivalents of *E*. *coli* T61 and T382), resulting in the accumulation of pEF-Tu on ribosomes and the consequent inhibition of translation (*8*).

Currently, there are two contrasting models that try to explain how phosphorylation inhibits the function of EF-Tu. The first model states that phosphorylation impairs ternary complex formation, which, in turn, precludes the interaction with the ribosome (*22*). This model is based on the analysis of the interaction of pEF-Tu with kirromycin in the context of GDP, GTP, and aa-tRNA. The authors predicted that, upon phosphorylation, the conformational landscape of the protein may be affected and that pEF-Tu adopts a new conformation different from that of GDP–GTP–aa-tRNA complexes (*22*). They argue that this new conformation could accelerate the kinetics of detachment of pEF-Tu from the ribosome compared to the nonphosphorylated protein. The second model states that the pEF-Tu–GTP complex stays bound to the ribosome because the GTPase activity is impaired (*8*). Ribosome pull-down assays in *B*. *subtilis* showed that pEF-Tu is enriched in the pellets containing 70*S* ribosomes, and the authors concluded that stabilization of the pEF-Tu–GTP–ribosome complex is what stalls translation (*8*). Both models put the effect of EF-Tu phosphorylation at a different step in translation and therefore seem mutually exclusive.

**Fig. 6:**
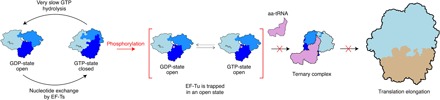
Regulation of the EF-Tu function in translation by phosphorylation. During translation, EF-Tu is observed in two predominant conformational states coupled to the nucleotide bound at a given moment. When bound to GTP, EF-Tu is in a closed state compatible with aa-tRNA binding (all three domains, shown in different shades of blue, are tightly packed). When bound to GDP, EF-Tu is in an open state with significantly weaker affinity for aa-tRNA binding (the three domains are more loosely arranged). This open form cannot usher aa-tRNAs to the ribosome for the elongation of protein synthesis. Phosphorylation (represented by a red dot) of EF-Tu traps the protein in its inactive open conformation independent of the nucleotide bound to the G domain. As shown in Fig. 1 (D and E), this decoupling of the conformational cycle from nucleotide binding precludes aa-tRNA binding and strongly inhibits translation. As in the case of the open GDP-bound form, phosphorylated EF-Tu is incompatible with aa-tRNA binding and cannot form a ternary complex to deliver aa-tRNAs to the ribosome (*21*). In this stable open state, subsequent phosphorylation might be facilitated, establishing a functional barrier preventing recoil from dormancy and other differentiation states.

Our data show that the inactivation of EF-Tu upon phosphorylation is based on the decoupling of nucleotide binding from the conformational cycle of the protein (Fig. 6). Phosphorylation traps EF-Tu in a conformational state that is closely related—if not identical—to the open form associated with GDP binding. In this form, GTP can be bound, but not hydrolyzed, and impaired dynamics also prevents binding of aa-tRNA and transferring to the translating ribosome. In this conformation, the affinity for ribosomes is likely to be weak as well and is difficult to reconcile with a stabilized ribosome–pEF-Tu–GTP complex. We have observed that phosphorylation also significantly decreases the solubility of EF-Tu, leading to aggregation in vitro. This might explain why, upon phosphorylation, pEF-Tu is found in pellets together with 70*S* ribosomes.

Phosphorylation of *E*. *coli* EF-Tu at T382 traps the protein in its inactive open conformation independent of the nucleotide bound to the G domain. The electronegative charge of the phosphate group of pT382 would strain the closed state of EF-Tu significantly, suggesting that the presence of a phosphate is incompatible with the closed active state. Using the phosphomimetic T61E mutant, we showed that the effect of phosphorylation is again to trap pEF-Tu_T61_ in an open inactive state or at least shift the dynamic equilibrium toward a dominant nonfunctional state. In this case, the phosphate group of pT61 interacts with K56, thereby stabilizing the β-hairpin conformation of switch I. As is the case of pT382, phosphorylation of T61 would lead to major clashes in the closed conformation of pEF-Tu_T61_, indicating that it is incompatible with the closed active state. EF-Tu is methylated and subsequently dimethylated at K56 upon entering the stationary phase in response to nutrient starvation (*4*). This perturbation of the switch I K56 by methylation is suggested to attenuate GTP hydrolysis and enhance translational accuracy (*3*).

Different phosphorylation sites with their distinct and overlapping functions and mechanisms not only allow fast stress responses but also seamlessly function as versatile epigenetic response systems, important for the adaptation of an organism to specific environments. In *Streptomyces*, ribosomal proteins are phosphorylated to attenuate translational activity when other ribosomal proteins are of low abundance, whereas in *Bacillus* phosphorylation is one of several dormancy switches (*8*, *35*). EF-Tu is highly conserved in bacteria, including the key phosphorylation sites T61 and T382 (*8*, *18*, *19*). Therefore, decoupling nucleotide binding from EF-Tu dynamics and the entrapment of pEF-Tu in a unique conformation that disrupts the translation cycle (Fig. 6) are likely general translation inhibition strategies among bacteria. After the initial phosphorylation event, which is sufficient to inactivate EF-Tu (with the protein trapped in the open form), subsequent phosphorylation might be facilitated, leading to the hyperphosphorylation of EF-Tu observed in bacterial cells under different conditions (*5*, *18*–*20*). This multisite phosphorylation could set a functional barrier preventing recoil from dormancy, cell cycle transitions, and other differentiation states observed in bacterial populations expressing phenotypic heterogeneity.

The functional significance of the decoupling of nucleotide binding from the EF-Tu conformational dynamic cycle may be relevant even in eukaryotes. The eukaryotic translation elongation factor eEF-1A2, an EF-Tu homolog, is AMPylated at a position structurally equivalent to *E*. *coli* T382 (*36*). In this case, a eukaryotic FIC enzyme (structural homolog of the bacterial kinase Doc but with adenylyltransferase activity) catalyzes the AMPylation reaction. One can easily imagine that the introduction of an AMP group at T382 would preclude the closed conformation, as does a phosphate group. This modification leads to translation inhibition and has a major effect in the survival of *Caenorhabditis elegans* upon *Pseudomonas* infection (*36*).

Overall, our results show that posttranslational modifications affecting the stability of the switch I region of EF-Tu are directly involved in tuning protein dynamics and are efficient allosteric switches. Moreover, phosphorylation is a very efficient conformational trap that keeps EF-Tu in an inactive open conformation, incompatible with translation. This conformational trap may be the linchpin behind further hyperphosphorylation observed during dormancy.

## MATERIALS AND METHODS

### Protein expression and purification

Wild-type Doc was purified as described previously (*37*–*40*). Cells transformed with a plasmid containing the *phd/doc* operon under a T7 promoter were grown in LB medium [supplemented with ampicillin (100 μg/ml)] at 310 K until the *A*_600_ was between 0.6 and 0.8. Expression of the genes was induced by adding 1 mM isopropyl-β-d-thiogalactopyranoside (IPTG). The cells were harvested by centrifugation 2 hours after induction and subsequently resuspended in 20 mM tris-HCl (pH 8.0), 1 mM EDTA, and a protease inhibitor cocktail. The cells were lysed with a cell disruptor at 277 K, and cell debris was removed by centrifugation. The cleared extract was loaded onto a Ni–nitrilotriacetic acid affinity column (connected to an ÄKTAexplorer FPLC system, GE Healthcare) pre-equilibrated in 20 mM tris-HCl (pH 8.0) and washed with the same buffer to remove nonbinding contaminants. To separate wild-type Doc from the antitoxin Phd, the procedure extensively described in previous studies (*37*–*40*) was followed.

His-tagged EF-Tu and the phosphomimetic mutants were purified on the basis of the protocol described by Castro-Roa *et al*. (*21*). Cells were grown in LB media supplemented with ampicillin (100 μg/ml) at 37°C until an *A*_600_ = 0.4 was reached. Protein expression was induced with IPTG (0.25 mM) at 30°C for 4 hours. After induction, cells were pelleted and washed twice with phosphorylation buffer (PB) containing 10 mM Hepes (pH 7.5), 60 mM NH_4_Cl, 10 mM Mg^2+^ acetate, and 2 mM tris(2-carboxyethyl)phosphine (TCEP). Pellets were resuspended in PB and EDTA-free protease inhibitor cocktail (Roche) and incubated on ice with lysozyme (0.1 mg/ml) for 30 min. Cells were disrupted by sonication in stainless steel tubes in an ice-water bath for 15 min, followed by two clearing centrifugation steps at 15,000 rpm in a JA-25.50 Beckman rotor. The supernatants were then applied onto a 5-ml HisTrap column (GE Healthcare) connected to an ÄKTAexplorer FPLC system (GE Healthcare). Elution was performed with a linear gradient of imidazole (from 10 to 200 mM) in 20 mM Hepes (pH 7.4) and 600 mM NaCl. Peak fractions were pooled and analyzed by SDS–polyacrylamide gel electrophoresis. Fractions containing EF-Tu were further purified by size exclusion chromatography (SEC) in PB. EF-Ts and all the EF-Tu mutants described in this work were produced and purified in the same way.

For the in vitro phosphorylation of EF-Tu, 1 mg of protein was incubated for 20 min at 37°C in the presence of 250 nM Doc and 250 μM adenosine triphosphate (ATP). The reaction buffer consisted of 50 mM Hepes (pH 7.5), 2 mM MgCl_2_, 40 mM NH_4_Cl, and 2 mM TCEP. After phosphorylation, pEF-Tu_T382_ was further purified by SEC.

### Native mass spectrometry

EF-Tu and pEF-Tu_T382_ samples were prepared at 10 μM protein concentration in 100 mM ammonium acetate buffer (pH 6.9). Both samples were introduced into the vacuum of the mass spectrometer using nanoelectrospray ionization with in-house–prepared gold-coated borosilicate glass capillaries with a voltage of approximately +1.4 kV. Spectra were recorded on a SYNAPT G2 HDMS Q-TOF (Waters) instrument. Critical voltages throughout the instrument were 50, 1, 25, 45, and 0.5 V for the sampling cone, extraction cone, trap collision voltage, trap dc bias, and transfer collision energy, respectively. Pressure throughout the instrument was 2.87 mbar for the source, 3.22 × 10^−2^ mbar for the trap collision cell, and 3.23 × 10^−2^ for the transfer collision cell. Mass spectra were externally calibrated using CsI, and the mass spectrometry data were analyzed using the MassLynx software version 4.1 (Waters).

### Isothermal titration calorimetry

ITC titrations were carried out on an Affinity ITC (TA Instruments). Before the measurement, nucleotide-free EF-Tu, pEF-Tu_T382_, and EF-Ts were dialyzed to 50 mM Hepes (pH 7.4), 40 mM NH_4_Cl, 2 mM MgCl_2_, and 1 mM TCEP. In each case, nucleotides were prepared with buffer from the last step of protein dialysis. The samples were filtered and degassed for 10 min before being examined in the calorimeter, and the titrations were performed at temperatures ranging from 10° to 35°C. All the experiments consisted of injection of constant volumes of 2 μl of titrant into the cell (200 μl) with a stirring rate of 75 rpm. Nominal sample concentrations were between 5 and 20 μM in the cell and 100 and 500 μM in the syringe. Actual sample concentrations were determined after dialysis or buffer exchange by measurement of their absorption at 280 nm. All data were analyzed using the MicroCal Origin ITC 7.0 and NanoAnalyze software packages. Binding affinities are reported in table S1.

### Analysis of the thermodynamic data

We performed ITC titrations at different temperatures to obtain the thermodynamic parameters describing the structural-energetic interplay of EF-Tu and pEF-Tu_T382_ with different ligands. The Gibbs-Helmholtz and Kirchhoff relations allow the calculation of the affinity of the interaction and the change in heat capacity upon binding (Δ*c*_p_, assumed to be constant across the range of experimental temperatures) as follows

$$\frac{d(\Delta G/T)}{dT}=\frac{\Delta H}{T^{2}}$$ (1)

$$\frac{d\Delta H}{dT}=\Delta c_{p}$$ (2)

From this analysis

ΔG=ΔH−TΔS (3)

$$\Delta G=-RT\text{ln}(1/K_{d})$$ (4)

$$\Delta H(T)=\Delta H_{r}(T_{r})+\Delta c_{p}(T-T_{r})$$ (5)

Δ*c*_p_ can be estimated from the slope of the plot of Δ*H*(*T*_i_) versus *T*_i_ . In addition, Δ*c*_p_ and Δ*H*_r_(*T*_r_) are empirically related to the polar (ΔASA_p_) and nonpolar (ΔASA_n_) solvent accessible surface area (ASA) of all participants, where Δ*H*_r_ is the enthalpy change at a reference temperature *T*_r_

$$\Delta H_{r}(T_{r})=a\cdot {\mathrm{\Delta ASA}}_{p}+b\cdot {\mathrm{\Delta ASA}}_{n}$$ (6)

$$\Delta c_{p}=c\cdot {\mathrm{\Delta ASA}}_{p}+d\cdot {\mathrm{\Delta ASA}}_{n}$$ (7)

*a* = 31.4 cal mol^−1^ Å^−2^, *b* = −8.44 cal mol^−1^ Å^−2^, *c* = −0.26 cal mol^−1^ K^−1^ Å^−2^, and *d* = 0.45 cal mol^−1^ K^−1^ Å^−2^ are empirical coefficients determined for the reference temperature *T*_r_ = 60°C, taken from Murphy and Freire (*41*) and Xie and Freire (*42*). The values for the buried area were converted into the amount of amino acids that were removed from the surface using the average ASA for apolar (34 Å^2^) and polar (56 Å^2^) amino acids (*43*). All the experimentally determined and calculated thermodynamic parameters are reported in table S1.

### Stopped-flow kinetics

Nucleotide binding kinetics were determined by fluorescence stopped-flow (SX18.MV; Applied Photophysics). All measurements were performed at 20°C in 50 mM tris (pH 7.5), 2 mM MgCl_2_, 40 mM NH_4_Cl, and 2 mM β-mercaptoethanol. EF-Tu or pEF-Tu_T382_ at a concentration of 1 μM was rapidly mixed with a concentration range (5 to 25 μM) of MANT-labeled nucleotides (MANT-GDP and MANT-GTPγS; Jena Bioscience). MANT-labeled nucleotides were excited indirectly at 280 nm using resonance energy transfer (FRET) between tryptophan and the MANT-labeled nucleotide, and the change in fluorescence was monitored through a 405-nm cutoff filter. For each nucleotide concentration, the data of at least five time traces were averaged and fitted to a single exponential function, yielding the observed rate constant *k*_obs_. The association rate constant *k*_on_ was obtained from the slope of the linear fit plotting the *k*_obs_ versus the nucleotide concentration. The dissociation rate constant *k*_off_ was obtained by mixing 200 μM unlabeled GDP with a mixture of 0.4 μM protein and 1.5 μM MANT-labeled nucleotide. The resulting time traces were fitted to a single exponential function. All measurements were performed as three independent repeats, and *k*_on_ and *k*_off_ values are given as means ± SD. The *K*_d_ values were calculated from the ratio of *k*_off_ and *k*_on_.

### [^3^H]Glu-tRNA^Glu^ incorporation

[^3^H]Glu-tRNA^Glu^ was prepared as described (*44*). The samples containing EF-Tu–GTP, EF-Tu–GDP, and pEF-Tu_T382_–GTP were transferred to ice, and 6 μl of [^3^H]Glu-tRNA^Glu^ (0.5 μM) was added to the mix to reconstitute the elongation complexes. After incubation at 0°C for 15 min, 3 μl of ribonuclease (RNase) A (at 0.7 mg/ml) was added and the reaction was incubated for 3 min. To quench RNase A activity, 5 μl of total tRNA was added followed by 40 μl of ice-cold 10% trichloroacetic acid (TCA). The reaction was filtered through a nitrocellulose filter (Bio-Rad) and washed six times with 200 μl of ice-cold 5% TCA. A final washing of 200 μl of 95% ethanol was performed, and the filters were allowed to dry. The filters were then transferred to scintillation vials containing 5 ml of scintillation cocktail, and the amount of [^3^H]Glu was determined by a scintillation counter machine.

### Fluorescent labeling

Oxygen was removed from the buffer [40 mM tris (pH 7.5), 40 mM (NH_4_)_2_SO_4_, 2 mM MgSO_4_] under vacuum and continuous stirring. TCEP was removed from the protein solution by gel filtration (PD-10 desalting columns, GE Healthcare Europe GmbH), and the protein was concentrated to at least 20 μM by ultrafiltration (nominal molecular weight limit = 10 kDa, Amicon Ultra-0.5, Merck Chemicals N.V.) at 6000*g* and 10°C. A sixfold molar excess of ATTO 488 maleimide (ATTO-TEC GmbH) and ninefold molar excess of Alexa Fluor 647 C_2_ maleimide (Life Technologies Europe BV) were mixed. The protein was added to a final concentration of at least 20 μM (preferentially 50 μM), and the samples were incubated for 2 hours at room temperature or overnight at 4°C. Unreacted dye was removed by gel filtration and ultrafiltration. A typical labeling result can be found in fig. S6A. The ATTO 488 maleimide typically had a 30 to 50% higher tendency for labeling the protein than the Alexa Fluor 647 maleimide. This could be due to the dye’s charge (ATTO 488, 1+; Alexa Fluor 647, 4−/1+), size (ATTO 488, 710 Da; Alexa Fluor 647, ~1250 Da), or structure. Between 20 and 50% of proteins typically carried two dyes after labeling. In our experience, this percentage depended on the protein concentration before labeling, the used dye batch, the degree of reduction of the Cys-SH moiety, and the time between protein purification and labeling. However, because FRET experiments are carried out using alternating FRET donor and acceptor excitation, the presence of each dye can be independently verified per passing molecule; the resulting FRET histograms thus present 100% of double-labeled molecules. Buffer containing 50% (w/v) glycerol was added 1:1, and the labeled protein sample was divided into aliquots and flash-frozen in liquid N_2_. However, because freezing/thawing unpredictably caused the appearance of a significant *E* = 0.9 to 1 FRET state, experiments were preferentially carried out on the freshly labeled, nonfrozen protein.

### FRET sample preparation

The labeled protein was diluted to 0.5 to 1 μM in buffer containing 10 mM of the nucleotide (GDP and GppNp from Jena Bioscience). The mixture was incubated for 2 hours at 37°C and further diluted at room temperature to 50 to 100 pM protein in buffer containing 1 mM nucleotide and bovine serum albumin (BSA) (0.1 mg/ml) (Sigma-Aldrich), and 30 μl was put on a coverslip (Nunc Lab-Tek Chambered Coverglass, Thermo Fisher Scientific BVBA) that was first incubated with BSA (1 mg/ml) and then washed twice with the sample solution. The background (needed for calculating *E* and *S* parameters, and for lifetime and PDA analysis) or scatter profile (needed for lifetime analysis) reference consisted of the same sample but without the protein or nucleotide. The small extra contribution of 1 mM nucleotide had a negligible effect. Measurements were performed at 22° or 37°C by placing the sample in a custom sample holder that was connected to a thermostatic water bath. When measurements were performed directly after mixing the protein and nucleotides, the closed/open ratio for the GDP state was higher. We attribute this to the presence of trace amounts of GTP in the GDP preparation. Therefore, we tested different premeasurement incubation conditions: overnight on ice and 2 hours at 37°C. We also tested whether measuring at 37°C instead of room temperature had an effect on the protein conformation.

### spFRET data recording

spFRET data were recorded on a homebuilt multiparameter fluorescence detection microscope with pulsed interleaved excitation (MFD-PIE) (*45*). Emission from a pulsed 483-nm laser diode (LDH-P-C-470, PicoQuant) was cleaned up (Chroma ET485/20x, F49-482; AHF analysentechnik AG), emission from a 635-nm laser diode (LDH-P-C-635B, PicoQuant) was cleaned up (Chroma z635/10x, PicoQuant), and both lasers were alternated at 26.67 MHz (PDL 828 Sepia II, PicoQuant), delayed ~18 ns with respect to each other, and combined with a 483-nm reflecting dichroic mirror in a single-mode optical fiber (coupler, 60FC-4-RGBV11-47; fiber, PMC-400Si-2.6-NA012-3-APC-150-P, Schäfter + Kirchhoff GmbH). After collimation (60FC-L-4-RGBV11-47, SuK GmbH), the linear polarization was cleaned up (CODIXX VIS-600-BC-W01, F22-601; AHF analysentechnik AG), and the light (100 μW of 483-nm light and 50 μW of 635-nm light) was reflected into the back port of the microscope (IX70, Olympus Belgium NV) and upward [3-mm-thick full-reflective Ag mirror, F21-005 (AHF) mounted in a total internal reflection fluorescence filter cube for BX2/IX2, F91-960; AHF analysentechnik AG] to the objective (UPLSAPO-60XW, Olympus). Sample emission was transmitted through a 3-mm-thick excitation polychroic mirror (Chroma zt470-488/640rpc, F58-PQ08; AHF analysentechnik AG), focused through a 75-μm pinhole (P75S, Thorlabs) with an achromatic lens (AC254-200-A-ML, Thorlabs), collimated again (AC254-50-A-ML, Thorlabs), and spectrally split (Chroma T560lpxr, F48-559; AHF analysentechnik AG). The blue range was filtered (Chroma ET525/50m, F47-525, AHF analysentechnik AG), and polarization was split (PBS251, Thorlabs). The red range was also filtered (Chroma ET705/100m, AHF analysentechnik AG), and polarization was split (PBS252, Thorlabs). Photons were detected on four avalanche photodiodes (PerkinElmer or EG&G SPCM-AQR12/14), which were connected to a time-correlated single-photon counting (TCSPC) device (SPC-630, Becker & Hickl GmbH) over a router (HRT-82, Becker & Hickl) and power supply (DSN 102, PicoQuant). Signals were stored in 12-bit first-in-first-out (FIFO) files. Microscope alignment was carried out using fluorescence correlation spectroscopy (FCS) on freely diffusing ATTO 488-CA and ATTO 655-CA (ATTO-TEC) and by connecting the detectors to a hardware correlator (ALV-5000/EPP) over a power splitter (PSM50/51, PicoQuant) for alignment by real-time FCS. Data were loaded in the PAM software (D. C. Lamb, Ludwig-Maximilians-Universität Munich) written in MATLAB (MathWorks). Instrument response functions (IRFs) were recorded in a solution of ATTO 488-CA or ATTO 655-CA in near-saturated centrifuged potassium iodide at a 25-kHz average count rate. Macrotime-dependent microtime shifting was present and corrected for two (blue/parallel and red/perpendicular) of four avalanche photodiodes (APDs) on the instrument response function (IRF) data. Signals from each TCSPC channel were divided in time gates to discern 483-nm excited FRET photons from 635-nm excited acceptor photons. A two-color MFD all-photon burst search algorithm (*46*) using a 500-μs sliding time window (minimum of 50 photons per burst, minimum of 5 photons per time window) and a kernel density estimator (ALEX-2CDE < 12) were used to identify single donor-acceptor–labeled molecules in the fluorescence trace.

### Multiparameter graphs

Per single molecule, a number of parameters were calculated. The corrected FRET efficiency was calculated as follows

$$E=\frac{F_{\text{BR}}-\alpha F_{\text{RR}}-\beta F_{\text{BB}}}{F_{\text{BR}}-\alpha F_{\text{RR}}-\beta F_{\text{BB}}+\gamma F_{\text{BB}}}$$ (8)

where *F*_BR_ = *S*_BR_ − *B*_BR_ is the background (*B*_BR_)–corrected number of photons in the red detection channel after blue excitation, *F*_BB_ = *S*_BB_ − *B*_BB_ is the background (*B*_BB_)–corrected number of photons in the blue detection channel after blue excitation, and *F*_RR_ = *S*_RR_ – *B*_RR_ is the background (*B*_RR_)–corrected number of photons in the red detection channel after red excitation. α is a correction factor for direct excitation of the FRET acceptor with the blue laser, β is a correction factor for cross-talk of the FRET donor in the red detection channel, and γ corrects for the differential detection efficiency in the blue and red detection channels.

The corrected stoichiometry was calculated as follows

$$S=\frac{F_{\text{BR}}-\alpha F_{\text{RR}}-\beta F_{\text{BB}}+\gamma F_{\text{BB}}}{F_{\text{BR}}-\alpha F_{\text{RR}}-\beta F_{\text{BB}}+\gamma F_{\text{BB}}+F_{\text{RR}}}$$ (9)

An invariant *S* parameter confirms a constant microscope and dye performance.

FRET donor lifetimes τ_D(A)_ and FRET acceptor lifetimes τ_A_ were determined using a burst-wise maximum likelihood estimator approach. Static FRET lines were calculated assuming an *R*_0_ = 53 Å, a donor-only lifetime τ_D_ (≈3.8 ns) estimated from the experimental data (stoichiometry > 0.8), and fast Gaussian distributed dye linker dynamics over 6 Å. Dynamic FRET lines were calculated using handpicked extreme state lifetimes as a reference.

The FRET donor anisotropy *r*_D_ and FRET acceptor anisotropy *r*_A_ were calculated from the burst-wise intensities in the different polarization channels. The anisotropy versus fluorescence lifetime plots were fitted with a Perrin equation

$$r=r_{0}/(1+t/q)$$ (10)

where *r* is the single-molecule anisotropy, *r*_0_ = 0.4 is the fundamental anisotropy, τ is the single-molecule fluorescence lifetime, and θ is the single-molecule rotational correlation time.

### Photon distribution analysis

Static PDA was carried out to obtain the absolute interdye distance distribution as described before, assuming two (or more) Gaussian distributed states (*47*). Dynamic PDA was carried out to obtain additional insights into conformational interconversion kinetics between these states, as described before (*48*). Practically, for each FRET data set, raw bursts were rebinned in different time bins (0.2, 0.5, 0.75, and 1 ms), and four histograms were constructed and analyzed simultaneously. Data were plotted in a FRET efficiency versus stoichiometry plot to deselect bins with complex acceptor photophysics, and only bins with at least 20 and maximally 250 photons (to reduce calculation time) were used for PDA analysis. A three-state model for a Gaussian distance distribution (*47*) was used to generate a library of simulated *E*_PR_ values, which was subsequently fitted to the experimental *E*_PR_ histogram using a reduced χ^2^–guided simplex search algorithm. When two data sets were compared [for example, EF-Tu(GDP) versus EF-Tu(GDPNP)], the eight histograms were analyzed simultaneously (as exemplified in fig. S6B): The mean and width of all Gaussian distributed substates were globally optimized over all samples, whereas the state area *A* (static PDA) or interconversion rate constants *k*_opening_ and *k*_closing_ (dynamic PDA) were globally optimized over a single sample. Moreover, the standard deviation σ of the distance distributions was globally optimized at a fraction *F* of the corresponding distance to increase fitting robustness, which has been shown before to be reasonable for FRET experiments with a blinking FRET acceptor (*49*, *50*). Finally, *F* was globally optimized over all states and data sets. We first validated this global fitting approach experimentally with a data set of nine conformationally static double-stranded DNA molecules with different D-A spacing (fig. S6, C and D). In summary, for a comparative analysis of two samples, only 10 parameters were needed to be optimized (*R*_1_, *R*_2_, *R*_static_, *F*, 2 × *k*_12_, 2 × *k*_21_, and 2 × *A*_3_). *R* is the FRET-averaged donor-acceptor distance 〈*R*_DA_〉_*E*_ (*50*). A probability density function (PDF) was calculated per state using the *R* and σ parameters obtained from PDA analysis that describe the underlying Gaussian distributed states. The summed PDF was scaled to a total area of unity, with the PDF area of each state scaled to the corresponding fraction of molecules. Criteria for a good fit were a low (<3) reduced χ^2^ value, as well as a weighted residuals plot free of trends. The relative 95% confidence intervals of the interconversion rate constants were calculated to estimate parameter determination robustness and were typically smaller than 25% for *k* values > 0.25 s^−1^. The resulting dwell times are presented as the average ± SD of at least three independent measurements originating from at least two protein purification batches. Correction parameters for PDA analysis were the average background count rates in donor and acceptor channels after donor excitation, donor cross-talk (β = 0.01), and the relative detection efficiency of donor and acceptor (γ = 0.69), both determined as described previously (*45*, *51*). Furthermore, we used a Förster radius of *R*_0_ = 53 Å that was calculated using the measured dye spectra, an orientation factor κ^2^ = 2/3, a measured quantum yield Φ = 0.62 for ATTO 488, and an extinction coefficient ε = 265,000 M^−1^ cm^−1^ for Alexa Fluor 647. The quantum yield was determined using a homebuilt absorbance/fluorescence spectroscope (*52*).

### spFRET simulation experiment to verify the range of interconversion rate constants

Details on the simulations: Simulation time was 2000 s in a 5-μm^3^ box containing 10 molecules diffusing at *D* = 1 μm^2^/s and a point spread function lateral waist *w*_r_ = 200 nm and axial waist *w*_z_ = 1000 nm. Fluorescence lifetimes and anisotropy were also simulated. During simulations, dye linkers were allowed to fluctuate over 5 Å. This averages out in a burst but is visible in the fluorescence lifetime. D-A distances were distributed Gaussian with a width σ = 5 Å, and a 10-ms static distance distribution rearrangement time was allowed. Simulations were performed at two signal-to-noise (SNR) ratios: high (to test whether the PDA fitting works in principle) and low (representative for the actual experiments) (table S5). Between *k* = 0.15 and 10/ms, dynamic PDA recovered the input parameters very well. Accuracy was marginally lower at low SNR (fig. S6 and table S6). In addition, the ratio of *k* values had to be <10 to allow significant population of each state.

### Crystallization and structure determination

For crystallization, nucleotide-free pEF-Tu_T382_, EF-Tu_T61E_, or EF-Tu_T382E_ (1 mg/ml) was mixed and incubated for 20 min at 37°C with 100 mM GDP, GTP, GTPγS, or GDPNP to prepare the different nucleotide complexes. Afterward, the samples were concentrated to approximately 10 mg/ml and filtered before use in the crystallization setup. Conditions were screened using a mosquito HTS robot from TTP Labtech (http://ttplabtech.com/).

Crystals of pEF-Tu_T382_ in complex with GDP grew after a few days in 0.2 M NaCl, 0.1 M MES (pH 6.5), and 10% PEG4000 (polyethylene glycol, molecular weight 4000). These crystals were cryoprotected by supplementing the mother liquor solution with 35% PEG4000 and directly vitrified in liquid N_2_ before data collection and diffracted to ~2.8 Å. The crystals of the EF-Tu_T61E_ and EF-Tu_T382E_ mutants with GDP grew in 0.1 M MgCl_2_, 0.1 M Hepes (pH 7.5), and 10% PEG4000. The crystals of pEF-Tu_T382_ in complex with GTP grew in 20% PEG3550, 0.1 M bis-tris propane (pH 6.5), and 0.2 M sodium iodide. Before data collection, these crystals were quick-soaked in 1 M sodium iodide (data collection in this case was done at 1.85 Å). For data collection, crystals were supplemented with 20% glycerol and vitrified in liquid N_2_.

In each case, data were processed with the XDS suite (*53*). In all cases, the unit-cell content was estimated with the program MATTHEW COEF from the CCP4 program suite (*54*). Molecular replacement was performed with Phaser (*55*). We split in individual domains the coordinates of the Protein Data Bank (PDB) structure 1EFC (*26*) and used them as a search model for molecular replacement. The molecular replacement solutions were then subjected to 30 cycles of normal refinement with BUSTER-TNT (*56*), which included rigid body and TLS refinement (as implemented in the RB and TLSBasic macros). In the later stages, refinement was implemented using the default options of the program. In the case of the complex of pEF-Tu_T382_ with GTP, the data were collected at a wavelength of 1.8369 Å and the structure was solved by MR-SAD using iodine also with Phaser (*55*). The crystal contained two molecules in the asymmetric unit, and 11 I atoms were placed with occupancy above 90%. The initial phases were of sufficient quality to allow automated building, performed with the ARP/wARP suite to almost completeness (~90% of the structure was built in this way). Structure refinement was performed with BUSTER-TNT (*56*) using the default options of the program. In each case, refinement was completed by combining manual building with Coot (*57*) and maximum likelihood refinement as implemented in BUSTER-TNT (*56*) using 5% of the reflections as a test set for cross-validation throughout the entire process. Structure quality was checked using MolProbity. Full data collection and refinement statistics are reported in table S3.

### Small-angle x-ray scattering

SAXS data were collected at the SWING and BM29 beamlines [Soleil and European Synchrotron Radiation Facility (ESRF) synchrotrons] using the standard beamline setup in SEC mode. The different protein samples were prepared as described above for crystallization in 25 mM Hepes (pH 7.5), 1 mM MgCl_2_, 1 mM TCEP, and 40 mM NH_4_Cl. Frames showing radiation damage were removed before data analysis. The data were analyzed with the ATSAS suite (*58*).

For SEC-coupled SAXS data collection, we used a Shodex KW402.5-4F column coupled to a high-performance liquid chromatography (HPLC) system, in front of the SAXS data collection capillary, to separate the excess noncomplexed material and thus remove this source of background. A flow rate of 0.2 ml/min was used, and the data were collected at 15°C. *Rg* values were obtained from the Guinier approximation and the Io by extrapolation to *q* = 0, as implemented in the ATSAS suite. This data collection strategy was extensively used to efficiently remove aggregates (which are a major problem for SAXS) while also allowing the collection of hundreds of scattering curves from the SEC peak that contains the sample of interest and a nearly perfect match with the reference buffer. The scattering curves, which cover a range of concentrations, were then compared and averaged to obtain the final data.

SAXS-based models were, in all cases, derived from the coordinates of crystal structures. The coordinates of each initial model were completed to account for missing loops and side chains using Modeller (*59*). Furthermore, all SAXS models were relaxed by molecular dynamics equilibration at 300 K (see below for further details), and sampling from the trajectory generated an initial ensemble of a few thousand models. We then used EOM (Ensemble Optimization Method) (*60*) and MES (Minimal Ensemble Search) (*61*) to select from this pool of structures the minimal ensembles with the best agreement with the experimental SAXS scattering curves. Figure S2 shows each ensemble together with the fitting of the calculated scattering curve from each ensemble to the experimental SAXS data. The calculation of ab initio shapes based on the scattering data was done with the program DAMMIF from the ATSAS package (*58*). Table S4 shows all the SAXS-derived parameters.

### Molecular dynamics simulations

The crystal structures of the EF-Tu–GDP (1EFC) and EF-Tu–GNP (1EXM) complexes were used as starting models for the simulation of EF-Tu in the open and closed conformations. The missing atoms from these structures were modeled using the program Modeller (*62*). The THP2 NAMD (*63*) patch was used to generate the different phosphorylated models. Furthermore, the GDP and GNP molecules were parameterized using the SwissParam server (www.swissparam.ch/).

The initial models were subjected to a conjugate gradient energy minimization in vacuum with the Cα restrained and then freed in a second minimization step. The complexes were then embedded in a water box, and electric neutrality was achieved by adding Na^+^ counter ions at 150 mM. The whole system was again energy-minimized in 3000 steps.

The molecular dynamics simulation was carried out for 1 ns with the program NAMD 2.7 (*63*) at constant temperature (310 K) and constant pressure (1 atm), with periodic boundaries and using CHARMM36 as force field. A time step of 2 fs was used to integrate the equations of motion. The short-range interactions were cut at 12 Å, and the smooth particle mesh Ewald method was used to calculate electrostatic interactions. Hydrogen atoms were constrained using the SHAKE algorithm. The VMD suite was used for the preparation of the system and for visualization and analysis of the trajectories.

### Site-directed mutagenesis

The oligonucleotides used for the construction of EF-Tu mutants are listed in table S9. pET-His–EF-Tu derivative plasmids carrying mutations were amplified using primers containing the mutations of interest and appropriate reverse primers using a Q5 polymerase (New England Biolabs) according to the manufacturer’s recommendations. Template DNA was removed by Dpn I treatment, and amplified fragments were phosphorylated using T4 PNK (New England Biolabs), ligated using T4 ligase (New England Biolabs), and transformed into *E*. *coli* MC1061 strain. The introduced mutations and the absence of secondary mutations were verified by sequencing of plasmid DNA. Plasmids were transformed into *E*. *coli* BL21(DE3) strain, and protein expression was confirmed by Western blotting with antibodies against the polyhistidine-tag (Sigma).

### Sequence analysis

A manually curated sequence alignment of the EF-Tu superfamily was retrieved from the Pfam database (*64*). The Pfam alignment was used as input for the program Skylign (*65*) to generate Logos representing the multiple sequence alignment and conservation of the superfamily. Figure S11 shows Logos illustrating the degree of conservation of each phosphorylation site.

## Supplementary Material

http://advances.sciencemag.org/cgi/content/full/4/3/eaap9714/DC1

## SUPPLEMENTARY MATERIALS

Supplementary material for this article is available at http://advances.sciencemag.org/cgi/content/full/4/3/eaap9714/DC1 table S1. Interplay between EF-Tu, pEF-Tu_T382_, and guanosine nucleotides. table S2. Parameters obtained from the stopped-flow kinetic measurements. table S3. X-ray data collection and refinement statistics. table S4. SAXS parameters of the different species. table S5. Further parameters obtained after PDA analysis of the experimental FRET data. table S6. Relevant input parameters for the spFRET simulations. table S7. Simulated spFRET parameters obtained after the dynamic PDA analysis. table S8. Solvent accessibility of experimentally validated phosphorylation sites in the *E*. *coli* proteome. table S9. The oligonucleotides used for the construction of the EF-Tu mutants EF-Tu_T382E_, EF-Tu_T61E_, EF-Tu_T382E/S222C_, and EF-Tu_T61E/S222C_. fig. S1. In vitro phosphorylation of EF-Tu by Doc. fig. S2. ITC titrations of EF-Tu and phosphorylated EF-Tu with nucleotides, EF-Ts and Glu-tRNA^Glu^. fig. S3. Stopped-flow kinetics of the EF-Tu and phosphorylated EF-Tu interaction with nucleotides. fig. S4. Interaction of EF-Tu and phosphorylated EF-Tu with aa-tRNAs. fig. S5. Structural effects of phosphorylation of EF-Tu at Thr^382^. fig. S6. Characterization of the labeling of EF-Tu with ATTO 488 and Alexa Fluor 647. fig. S7. Multiparameter graphs obtained after spFRET analysis of EF-Tu and phosphorylated EF-Tu in the presence of GDP and GDPNP. fig. S8. Multiparameter graphs obtained after spFRET analysis of the EF-Tu phosphomimetic mutants in the presence of GDP and GDPNP. fig. S9. Static versus dynamic PDA analysis of the EF-Tu phosphomimetic mutants in the presence of GDP and GDPNP. fig. S10. X-ray structure of the EF-Tu phosphomimetic mutants. fig. S11. Analysis of the conservation of the phosphorylation sites across the EF-Tu superfamily.
