# Supplementary material for: Phosphorylation decelerates conformational dynamics in bacterial translation elongation factors
Source: Sci Adv. 2018 Mar 14;4(3):eaap9714. doi: 10.1126/sciadv.aap9714 (PMC5851678; doi:10.1126/sciadv.aap9714)
Supplement: http://advances.sciencemag.org/cgi/content/full/4/3/eaap9714/DC1 [file supp_4_3_eaap9714__index.html]

Science Advances | Science Advances

## Supplementary Materials

**This PDF file includes:**

- table S1. Interplay between EF-Tu, pEF-TuT382, and guanosine nucleotides.
- table S2. Parameters obtained from the stopped-flow kinetic measurements.
- table S3. X-ray data collection and refinement statistics.
- table S4. SAXS parameters of the different species.
- table S5. Further parameters obtained after PDA analysis of the experimental FRET data.
- table S6. Relevant input parameters for the spFRET simulations.
- table S7. Simulated spFRET parameters obtained after the dynamic PDA analysis.
- table S8. Solvent accessibility of experimentally validated phosphorylation sites in the *E. coli* proteome.
- table S9. The oligonucleotides used for the construction of the EF-Tu mutants EF-TuT382E, EF-TuT61E, EF-TuT382E/S222C, and EF-TuT61E/S222C.
- fig. S1. In vitro phosphorylation of EF-Tu by Doc.
- fig. S2. ITC titrations of EF-Tu and phosphorylated EF-Tu with nucleotides, EF-Ts and Glu-tRNAGlu.
- fig. S3. Stopped-flow kinetics of the EF-Tu and phosphorylated EF-Tu interaction with nucleotides.
- fig. S4. Interaction of EF-Tu and phosphorylated EF-Tu with aa-tRNAs.
- fig. S5. Structural effects of phosphorylation of EF-Tu at Thr382.
- fig. S6. Characterization of the labeling of EF-Tu with ATTO 488 and Alexa Fluor 647.
- fig. S7. Multiparameter graphs obtained after spFRET analysis of EF-Tu and phosphorylated EF-Tu in the presence of GDP and GDPNP.
- fig. S8. Multiparameter graphs obtained after spFRET analysis of the EF-Tu phosphomimetic mutants in the presence of GDP and GDPNP.
- fig. S9. Static versus dynamic PDA analysis of the EF-Tu phosphomimetic mutants in the presence of GDP and GDPNP.
- fig. S10. X-ray structure of the EF-Tu phosphomimetic mutants.
- fig. S11. Analysis of the conservation of the phosphorylation sites across the EF-Tu superfamily.

Download PDF

**Files in this Data Supplement:**

- Adobe PDF - aap9714\_SM.pdf
