# Supplementary material for: Phosphorylation decelerates conformational dynamics in bacterial translation elongation factors
Source: Sci Adv. 2018 Mar 14;4(3):eaap9714. doi: 10.1126/sciadv.aap9714 (PMC5851678; doi:10.1126/sciadv.aap9714)
Supplement: http://advances.sciencemag.org/cgi/content/full/4/3/eaap9714/DC1 [file aap9714_SM.pdf]

Science Advances

AAAS

advances.sciencemag.org/cgi/content/full/4/3/eaap9714/DC1

Supplementary Materials for

Phosphorylation decelerates conformational dynamics in bacterial translation elongation factors

Ariel Talavera, Jelle Hendrix, Wim Versées, Dukas Jurėnas, Katleen Van Nerom, Niels Vandenberk, Ranjan Kumar Singh, Albert Konijnenberg, Steven De Gieter, Daniel Castro-Roa, Anders Barth, Henri De Greve, Frank Sobott, Johan Hofkens, Nikolay Zenkin, Remy Loris, Abel Garcia-Pino

Published 14 March 2018, *Sci. Adv.* **4**, eaap9714 (2018)
DOI: 10.1126/sciadv.aap9714

This PDF file includes:

- table S1. Interplay between EF-Tu, pEF-Tu$_{T382}$, and guanosine nucleotides.
- table S2. Parameters obtained from the stopped-flow kinetic measurements.
- table S3. X-ray data collection and refinement statistics.
- table S4. SAXS parameters of the different species.
- table S5. Further parameters obtained after PDA analysis of the experimental FRET data.
- table S6. Relevant input parameters for the spFRET simulations.
- table S7. Simulated spFRET parameters obtained after the dynamic PDA analysis.
- table S8. Solvent accessibility of experimentally validated phosphorylation sites in the *E. coli* proteome.
- table S9. The oligonucleotides used for the construction of the EF-Tu mutants EF-Tu$_{T382E}$, EF-Tu$_{T61E}$, EF-Tu$_{T382E/S222C}$, and EF-Tu$_{T61E/S222C}$.
- fig. S1. In vitro phosphorylation of EF-Tu by Doc.
- fig. S2. ITC titrations of EF-Tu and phosphorylated EF-Tu with nucleotides, EF-Ts and Glu-tRNA^{Glu}.
- fig. S3. Stopped-flow kinetics of the EF-Tu and phosphorylated EF-Tu interaction with nucleotides.
- fig. S4. Interaction of EF-Tu and phosphorylated EF-Tu with aa-tRNAs.
- fig. S5. Structural effects of phosphorylation of EF-Tu at Thr^{382}.
- fig. S6. Characterization of the labeling of EF-Tu with ATTO 488 and Alexa Fluor 647.
- fig. S7. Multiparameter graphs obtained after spFRET analysis of EF-Tu and phosphorylated EF-Tu in the presence of GDP and GDPNP.

- fig. S8. Multiparameter graphs obtained after spFRET analysis of the EF-Tu phosphomimetic mutants in the presence of GDP and GDPNP.
- fig. S9. Static versus dynamic PDA analysis of the EF-Tu phosphomimetic mutants in the presence of GDP and GDPNP.
- fig. S10. X-ray structure of the EF-Tu phosphomimetic mutants.
- fig. S11. Analysis of the conservation of the phosphorylation sites across the EF-Tu superfamily.

Supplementary Information

Supplementary Tables

table S1. Interplay between EF-Tu, pEF-Tu$_{T382}$, and guanosine nucleotides. The binding affinities were determined from fitting a single interaction models to the experimental ITC thermograms according to the experimental setup (see the main text and the Supplementary Material and Methods for details). Data represent mean values ± s.d. (*).

Titrations	ΔG (kcal·mol^{-1})	ΔH (kcal·mol^{-1})	ΔS (cal·K^{-1}·mol^{-1})	Kd (nM)	Δcp (kcal·K^{-1}·mol^{-1})	ΔASAp	ΔASAn	Interface residues	Temp (°C)	# of exp
**EF-Tu + GDP**	-9.8 ± 0.1<br>-11.0 ± 0.3<br>-12.1 ± 0.1	-4.9 ± 0.4<br>-6.2 ± 0.5<br>-6.3 ± 0.3	16.9 ± 0.8<br>17 ± 1<br>19 ± 1	36.5 ± 0.2<br>4.5 ± 0.6<br>1.4 ± 0.2	-0.13 ± 0.02	-498.3	-576.8	25 (16)	15<br>20<br>25	3<br>3<br>4
**EF-Tu + GTPγS**	-9.1 ± 0.1<br>-9.68 ± 0.05<br>-10.2 ± 0.1	-0.5± 0.3<br>-1.1 ± 0.6<br>-5.0 ± 0.5	29.9 ± 0.5<br>29.6 ± 0.7<br>17 ± 1	130 ± 5<br>81 ± 6<br>59.0 ± 0.9	-0.23 ± 0.03	-570.2	-840.6	32 (28)	15<br>20<br>35	3<br>4<br>3
**pEF-Tu$_{T382}$ + GDP**	-9.0 ± 0.1<br>-11 ± 0.4<br>-11.99 ± 0.01	-5.6 ± 0.5<br>-6.5 ± 0.4<br>-6.9 ± 0.7	11.7 ± 0.8<br>15 ± 1<br>17 ± 2	146 ± 1<br>5.7 ± 0.5<br>1.64 ± 0.02	-0.135 ± 0.005	-525.4	-592.5	26 (18)	15<br>20<br>25	3<br>3<br>3
**pEF-Tu$_{T382}$ + GTPγS**	-7.7 ± 0.1<br>-9.45 ± 0.05<br>-9.7 ± 0.1	-4.0 ± 0.2<br>-5.1 ± 0.1<br>-5.2 ± 0.2	12.7 ± 0.5<br>14.9 ± 0.3<br>15 ± 1	1530 ± 10<br>90 ± 8<br>74.5 ± 0.5	-0.12 ± 0.03	-439.3	520.5	22	15<br>20<br>25	3<br>3<br>3
**EF-Tu + GDPNP**	-9.0 ± 0.3	-1.8 ± 0.3	25 ± 1	128 ± 8					20	3
**pEF-Tu$_{T382}$ + GDPNP**	-8.7 ± 0.2	-4.1 ± 0.5	15.6 ± 0.9	320 ± 1					20	3
**EF-Tu + EFTs**	-12.2 ± 0.1	-1.3± 0.4	36 ± 1	1.2± 0.2					25	2
**pEF-Tu$_{T382}$ + EFTs**	-12.5 ± 0.2	-6.6 ± 0.5	19.6 ± 0.9	0.8 ± 0.3					25	2
**EF-Tu: GTPγS + E-tRNA^{E}**	-10.8 ± 0.4	-4.5± 0.8	21 ± 2	12 ± 4					25	2

table S2. Parameters obtained from the stopped-flow kinetic measurements. k_{on} is determined as the slope of the linear plot of the k_{obs} value versus the nucleotide concentration; k_{off} as the rate constant of protein-nucleotide dissociation (see fig. S3). Data represent mean values ± s.d. (n=3 independent repeats). K_d is calculated as the ratio between k_{off} and k_{on}.

Titrations	***kon*** **(μM^{-1} s^{-1})**	***koff*** **(s^{-1})**	**Kd (nM)**
**EF-Tu + GDP**	2.5 ± 0.3	0.0017 ± 0.0001	0.7 ± 0.1
**EF-Tu + GTPγS**	0.46 ± 0.03	0.038 ± 0.0012	81 ± 6
**pEF-Tu$_{T382}$ + GDP**	2.6 ± 0.1	0.0016 ± 0.0001	0.61 ± 0.04
**pEF-Tu$_{T382}$ + GTPγS**	0.52 ± 0.03	0.0397 ± 0.0012	76 ± 5

table S3. X-ray data collection and refinement statistics. Parameters for the highest resolution shell are between brackets.

Data set	pEF-Tu$_{T382}$-GDP	pEF-Tu$_{T382}$-GTP	EF-Tu$_{T382E}$-GDP	EF-Tu$_{T61E}$-GDP
Beamline	Proxima 1 (Soleil Paris)	Proxima 2A (Soleil Paris)	i24 (DLS, Oxfordshire)	Proxima 2A (Soleil Paris)
Wavelength (Å)	1.000	1.8369	0.8696	0.9801
Space group	$P2_12_12$	P1	P1	P1
Unit cell a (Å)	68.2	59.8	55.9	58.1
Unit cell b (Å)	244.4	61.9	61.5	62.1
Unit cell c (Å)	61.7	65.5	65.0	65.5
Unit cell α (°)	90.0	70.4	71.6	109.3
Unit cell β (°)	90.0	75.8	71.2	106.0
Unit cell γ (°)	90.0	89.4	87.8	88.1
Resolution limits (Å)	45.8 – 2.80 (2.87 – 2.80)	51.4 – 2.81 (2.86 – 2.81)	52.8 – 2.18 (2.24 – 2.18)	51.0 – 3.29 (3.50 – 3.29)
Number of measured reflections	184750 (12183)	119677 (3374)	131124 (9845)	20766 (3481)
Number of unique reflections	25555 (2274)	20019 (950)	38398 (2828)	11351 (1854)
Multiplicity	7.2 (5.4)	6.0 (3.6)	3.4 (3.5)	1.8 (1.9)
Completeness	97.1 (89.0)	96.6 (93.4)	90.0 (86.0)	98.0 (91.7)
Rmerge (%)	13.4 (22.8)	11.2 (28.9)	10.5 (99.0)	14.7 (85.0)
$\langle I/\sigma(I)\rangle$	19.4 (8.0)	9.0 (2.4)	8.3 (1.6)	6.2 (1.4)
$CC_{1/2}$	1.0 (0.97)	0.98 (0.93)	1.0 (0.5)	0.99 (0.63)
R-factor (%)	15.4	18.1	18.7	22.5
R_{free}-factor (%)	19.8	25.8	22.5	24.7
Ramachandran profile				
Core	98.0	96.7	96.2	96.0
Other allowed	2.0	3.1	3.8	4.0
Outliers	0.0	0.1	0.0	0.0
R.m.s. deviations				
Bond lengths (Å)	0.008	0.010	0.014	0.010
Bond angles (°)	1.027	1.29	1.97	2.90
Number of atoms	6153	6070	6030	5282
Protein	5851	5654	5714	5213
Ligands	58	105	58	58
Water	244	311	201	11
Other	-	-	57	-
B-factors (Å^2)				
From Wilson plot	37.8	18.5	50.9	99.3
All atoms	42.0	20.0	57.3	111.6
Protein atoms	42.4	20.3	56.8	111.8
Water atoms	34.5	13.4	62.4	32.0
Other atoms	29.0	23.9	73.6	111.5
PDB entry	5MI3	5OPD	5MI8	5MI9

table S4. SAXS parameters of the different species. The quality of the SAXS-based models was assessed based on the metrics recently proposed by Rambo and Tainer which make use of the *s(I(s))* vs. *s* invariant. Theoretical and calculated values are shown in brackets.

Sample	***Rg* (Å)**	**D_{max} (Å)**	**MW (kDa)**	χ^2	**R_{SAS}**	**Vc**
**EF-Tu:GTPγS**	24.7 (23.7)	72	46.0 (44.7)	0.8	0.0016	373.9 (372.5)
**EF-Tu: GDPNP**	24.7 (23.7)	76	44.9 (44.7)	0.6	0.0020	371 (372.5)
**EF-Tu:GDP**	26.3 (25.4)	84	47.0 (44.6)	1.6	0.0027	400 (383.9)
**pEF-Tu$_{T382}$:GTPγS**	26.0 (25.6)	83	44.2 (44.6)	0.7	0.0005	379 (383.9)
**pEF-Tu$_{T382}$: GDPNP**	26.3 (25.6)	85	45.2 (44.8)	0.9	0.0007	382.9 (383.9)
**pEF-Tu$_{T382}$:GDP**	26.7 (25.4)	85	43.5 (44.7)	0.4	0.0026	378.5 (383.9)

table S5. Further parameters obtained after PDA analysis of the experimental FRET data. The indicated χ^2 values are the average of four datasets, corresponding to the four analysed time windows. $R_{open/closed}$ is the FRET-averaged distance $\langle R_{DA} \rangle_E$ between donor and acceptor dyes and was optimized globally for all datasets, unless explicitly indicated otherwise. The $k_{opening/closing}$ parameters are in Fig. 4g. *F* is the fraction of *R* needed to describe broadening of the FRET states due to static heterogeneity or fast (<< 1 ms) dynamic heterogeneity. *F* was globally optimized for all states in the 8 datasets, unless explicitly indicated otherwise. [a]Values for *R* were optimized separately for each nucleotide condition. [c]Essentially, the fit evolved to *k*-values equal to 0. [b]For the static PDA analysis of the T61E data, the GDP and GDPNP datasets were analyzed separately. [c]The fit quality was adequate because the lack of dynamics in the model was wrongfully compensated by altered values for *R*.

Sample	Dynamic model				Static model
	mean χ^2	R_{open} (Å)	R_{closed} (Å)	F (%)	mean χ^2
**EF-Tu:GDP**	1.43	50.2	34.9	10	3.39
**EF-Tu:GDPNP**	1.77				4.13
**pEF-Tu$_{T382}$:GDP**	2.01	51.4	34.4	9	2.11
**pEF-Tu$_{T382}$:GDPNP**	1.57				1.70
**EF-Tu$_{T382E}$:GDP**	4.27	52.0	35.8	9	5.26[a]
**EF-Tu$_{T382E}$:GDPNP**	4.43				2.61[a]
**EF-Tu$_{T61E}$:GDP**	2.24	50.6	34.7	9	2.02[b]
**EF-Tu$_{T61E}$:GDPNP**	1.96				1.39[b,c]

table S6. Relevant input parameters for the spFRET simulations.

	high SNR	low SNR
F_1 [kHz]	1000	200
F_2 [kHz]	1000	100
B_1 [kHz]	0	0.87
B_2 [kHz]	0	0.87
R_0 [Å]	50	50

F is the peak brightness of a molecule when diffusing through the focus. *B* is a constant uncorrelated noise contribution.

table S7. Simulated spFRET parameters obtained after the dynamic PDA analysis.

Static FRET simulation	R_1 [Å]	σ_1 [Å]	R_2 [Å]	σ_2 [Å]	k_{12} [/ms]	t_1 [ms]	k_{21} [/ms]	t_2 [ms]
input parameters	40	5	60	5	/	/	/	/
dynamic PDA, high SNR	40.9	4.3	59.1	4.9	0.02	50.00	0.01	100.00
dynamic PDA, low SNR	41.2	4.7	59.4	4.2	0	/	0.00	/

Dynamic FRET simulation	R_1 [Å]	σ_1 [Å]	R_2 [Å]	σ_2 [Å]	k_{12} [/ms]	t_1 [ms]	k_{21} [/ms]	t_2 [ms]
input parameters	40	5	60	5	0.05	20.00	0.25	4.00
dynamic PDA, low SNR	40.9	4.5	58.5	4.7	0	/	0.00	/
input parameters					0.15	6.67	0.15	6.67
dynamic PDA, low SNR	40.8	4.4	59.2	4.9	0.18	5.56	0.18	5.56
input parameters					0.15	6.67	0.50	2.00
dynamic PDA, high SNR	40.7	4.4	58.7	4.9	0.15	6.67	0.48	2.08
dynamic PDA, low SNR	40.8	4.4	59.8	4.1	0.17	5.88	0.61	1.64
input parameters					0.5	2.00	0.50	2.00
dynamic PDA, high SNR	40.7	4.44	59.1	4.7	0.54	1.85	0.53	1.89
dynamic PDA, low SNR	40.6	4.2	58.8	4.6	0.57	1.75	0.54	1.85
input parameters					1	1.00	0.50	2.00
dynamic PDA, high SNR	41.1	4.63	59.29	4.62	1.02	0.98	0.52	1.92
dynamic PDA, low SNR	41.4	4.6	59.5	4.8	1.08	0.93	0.56	1.79
input parameters					2	0.50	0.50	2.00
dynamic PDA, high SNR	40.7	4.4	59.2	4.7	2.14	0.47	0.52	1.92
dynamic PDA, low SNR	39.9	3.7	58.9	4.9	2.29	0.44	0.48	2.08
input parameters					2	0.50	2.00	0.50
dynamic PDA, low SNR*	40.6	4.2	58.3	4.8	2.26	0.44	1.99	0.50
input parameters					10	0.10	10.00	0.10
dynamic PDA, low SNR*	39.8	4.4	58.4	5	13.6	0.07	10.90	0.09

/ parameter was not included or could not be calculated. * initial guess had to be good. $t = 1/k$ is the dwell time in a state.

table S8. Solvent accessibility of experimentally validated phosphorylation sites in the *E. coli* proteome. Phosphorylation sites that become exposed or increase solvent accessibility by 25% or more as a result of the conformational change coupled to GTP hydrolysis are shown in bold.

EF-Tu phospho-peptides	site	domain
ARGI***pT***INTSHVEY	**61**	G-domain
ARGITIN***pT***SHVEY	64	G-domain
ARGITINT***pS***HVEY	65	G-domain
ELL***pS***QYDFP	157	G-domain
DD***pT***PIVRG	167	G-domain
PIVRG***pS***AL	174	G-domain
DVFSI***pS***GR	**219**	β-barrel I
FESEVYIL***pS***K	312	β-barrel II
GYRPQF***pY***FR	**331**	β-barrel II
GGR***pT***VGA	**382**	β-barrel II

table S9. The oligonucleotides used for the construction of the EF-Tu mutants EF-Tu$_{T382E}$, EF-Tu$_{T61E}$, EF-Tu$_{T382E/S222C}$, and EF-Tu$_{T61E/S222C}$.

Primer	Sequence 5’-3’
R-Tu-mutS222C	GCAGATGGAGAATACGTCTTCGATCGG
F-Tu-S222C	GGTCGTGGTACCGTTGTTACCGG
F-Tu-T61E	ATCAACACTTCTCACGTTGAAT
R-Tu-mutT61E	TTCGATACCACGAGCTTTTTCTTCC
F-Tu-T382E	GTTGGCGCGGGCGTTGTTG
R-Tu-mutT382E	TTCACGGCCGCCTTCACGGATT

Supplementary Figures

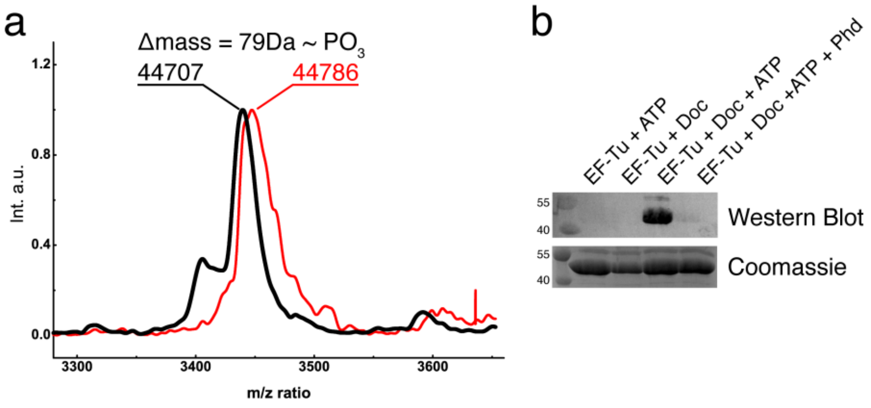

fig. S1. In vitro phosphorylation of EF-Tu by Doc. (**a**) Native mass spectrum of EF-Tu in black (44707 Da) and EF-Tu incubated with ATP and the toxin Doc in red (44786). The difference in mass between the peak corresponding to EF-Tu and pEF-TuT382 is 79 Da, which is in the range of the expected average increase in mass due to phosphorylation (79.97 Da). (**b**) Coomassie-stained SDS-PAGE and Western blot of purified EF-Tu treated and non-treated with the kinase Doc and ATP. The Western blot was performed using an antibody specific against phospho-threonine peptides and reveals that Doc specifically modifies EF-Tu at a threonine site as shown previously[42].

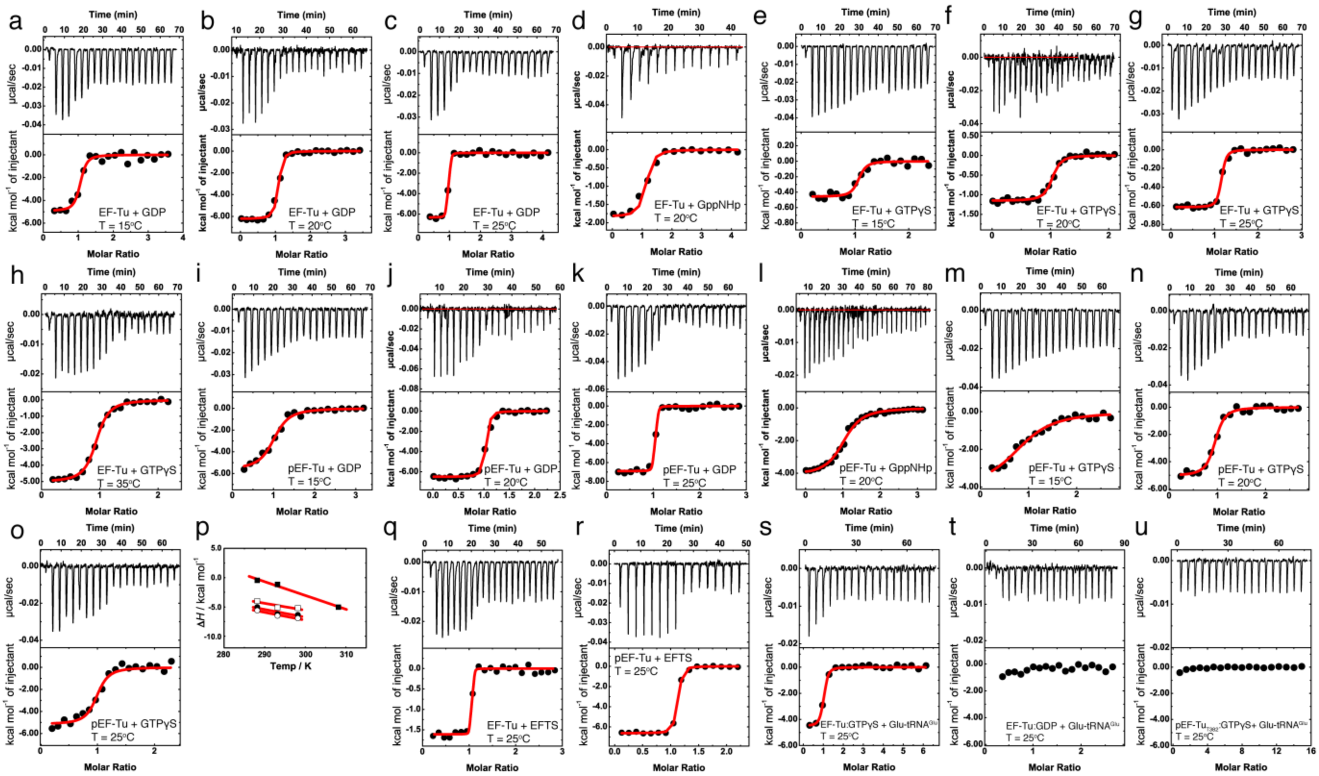

fig. S2. ITC titrations of EF-Tu and phosphorylated EF-Tu with nucleotides, EF-Ts and Glu-tRNA^{Glu}. ITC titrations of EF-Tu with GDP at 15 °C (**a**), 20 °C (**b**), 25 °C (**c**); EF-Tu with GDPNP at 20 °C (**d**); EF-Tu with GTPγS at 15 °C (**e**), 20 °C (**f**), 25 °C (**g**), 35 °C (**h**); pEF-Tu$_{T382}$ with GDP at 15 °C (**i**), 20 °C (**j**), 25 °C (**k**); pEF-Tu$_{T382}$ with GDPNP at 20 °C (**l**); pEF-Tu$_{T382}$ with GTPγS at 15 °C (**m**), 20 °C (**n**), 25 °C (**o**); (**p**) temperature dependency of binding enthalpy changes (ΔH) upon EF-Tu and pEF-Tu$_{T382}$ binding to GDP and GTPγS (see table S1 for details). ITC titrations of EF-Tu with EF-TS (**q**) and pEF-Tu$_{T382}$ EF-TS (**r**). ITC titrations of EF-Tu-GTPγS with Glu-tRNA^{Glu} (**s**), EF-Tu-GDP with Glu-tRNA^{Glu} (**t**) and pEF-Tu-GTPγS with Glu-tRNA^{Glu} (**u**). Table S1 contains all the thermodynamic parameters of each titration.

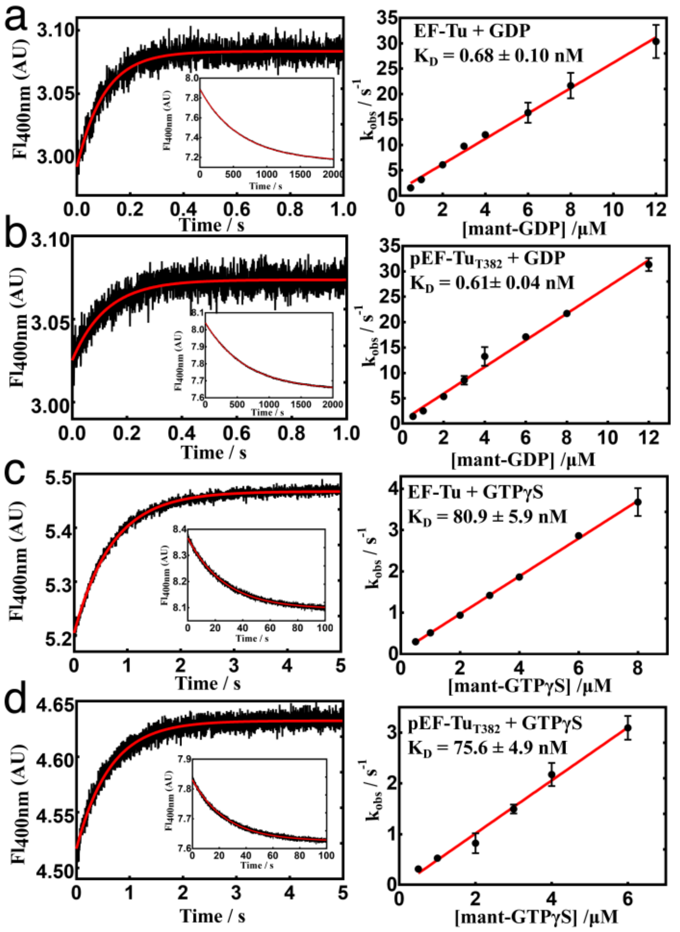

fig. S3. Stopped-flow kinetics of the EF-Tu and phosphorylated EF-Tu interaction with nucleotides. Kinetics of guanine nucleotide binding and dissociation to EF-Tu and pEF-Tu$_{T382}$ monitored by stopped flow. The interaction was measured by FRET excitation of mant fluorescence upon mixing 1 µM of protein with increasing concentrations of mant-nucleotide. Representative time courses of formation of mant-GDP:EF-Tu (**a**), mant-GDP: pEF-Tu$_{T382}$ (**b**), mant-GTPγS:EF-Tu (**c**), and mant-GTPγS:pEF-Tu$_{T382}$ (**d**). Inset: the dissociation of each complex upon rapid mixing with an excess (200 µM) of unlabeled GDP. The panels to the right of (a-d) show the concentration dependence of the observed rate constant (k_{obs}) of mant-GDP:EF-Tu, mant-GDP:pEF-Tu$_{T382,}$ mant-GTPγS:EF-Tu, and mant-GTPγS:pEF-Tu$_{T382}$ complex formation. Each data point represents the average (± s.d.) of three independent measurements. K_d is determined as the ratio of k_{off} and k_{on}.

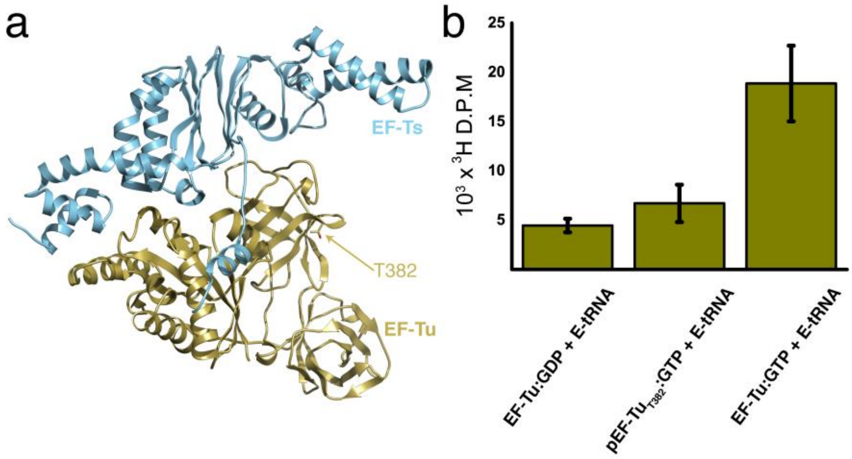

fig. S4. Interaction of EF-Tu and phosphorylated EF-Tu with aa-tRNAs. (**a**) Structure of the EF-Tu-GDP:EF-Ts complex. The position of EF-Tu T382 is highlighted in the figure. Considering that T382 is away from the EF-Tu:EF-Ts interaction site is not surprising phosphorylation does not affect the interaction between both translation factors. (**b**) Binding EF-Tu and pEF-TuT382 to Glu-$tRNA^{Glu}$). EF-Tu-GTP and pEF-Tu_{T382}-GTP were mixed $^{[3H]-}$Glu-$tRNA^{Glu}$ (0.5 uM) and incubated in ice for 15 min. The figure shows that in the complex with GDP or phosphorylated EF-Tu there is less incorporation of [3H] compared to the EF-Tu-GTP complex.

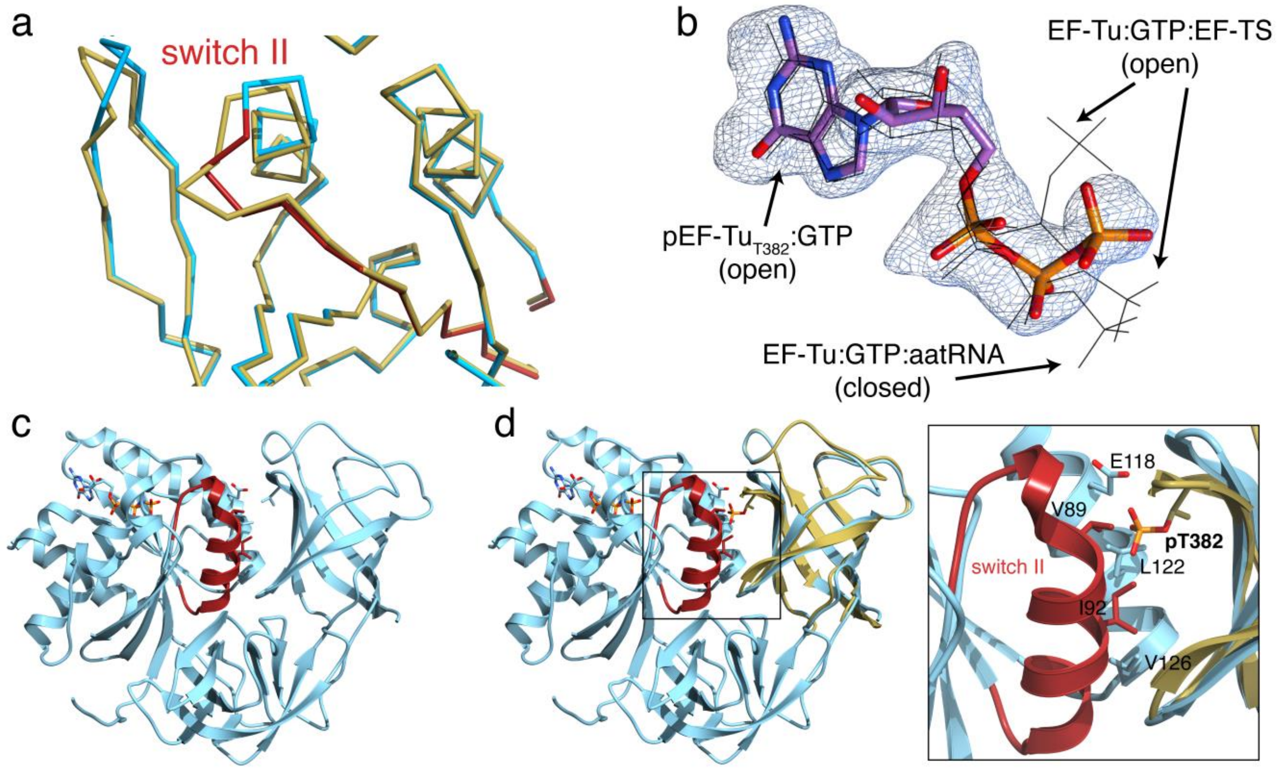

fig. S5. Structural effects of phosphorylation of EF-Tu at Thr^{382}. (**a**) Stereo view of the superposition of the Cα-atoms of the crystal structure of pEF-Tu$_{T382}$ bound to GDP (in cyan) on the structure of the non-phosphorylated EF-Tu bound to GDP (in ochre). The 5°−rotation of the β-barrel I domain relative to the G-domain and β-barrel II is highlighted in the figure. (**b**) Details of the structural rearrangements in the crucial switch I and switch II of EF-Tu induced by the phosphorylation at T382. Cα-atoms are colored as in (**a**). Closed state of EF-Tu, all 3 domains of EF-Tu contributes to a large contact interface that results in globular shape in stark contrast with the structure of the open state. The center of the closed shape where all three domains coalesce plays a major role in the binding to amino acyl tRNAs. (**c**) EF-Tu-GTP complex in the closed state, the switch II is shown in red together with the aliphatic side chains that communicate both switch regions. (**d**) The β-barrel II domain from the structure of pEF-TuT382 bound to GDP (in ochre) is shown superimposed on the structure of the EF-Tu-GTP complex, the panel on the right shows the details of the superposition at the interface between the switch II and the phosphorylated T382 from β-barrel II. From the analysis of the superposition it becomes apparent that the addition of a phosphate group would lead to sever clashes in the interface dominated by hydrophobic interaction, likely precluding the closed state once the protein is phosphorylated. (**e**) Different conformational states observed for GTP (or GDPNP) when bound to EF-Tu (all shown in black), superimposed on the conformation observed in the crystal structure of the pEF-Tu$_{T382}$–GTP complex.

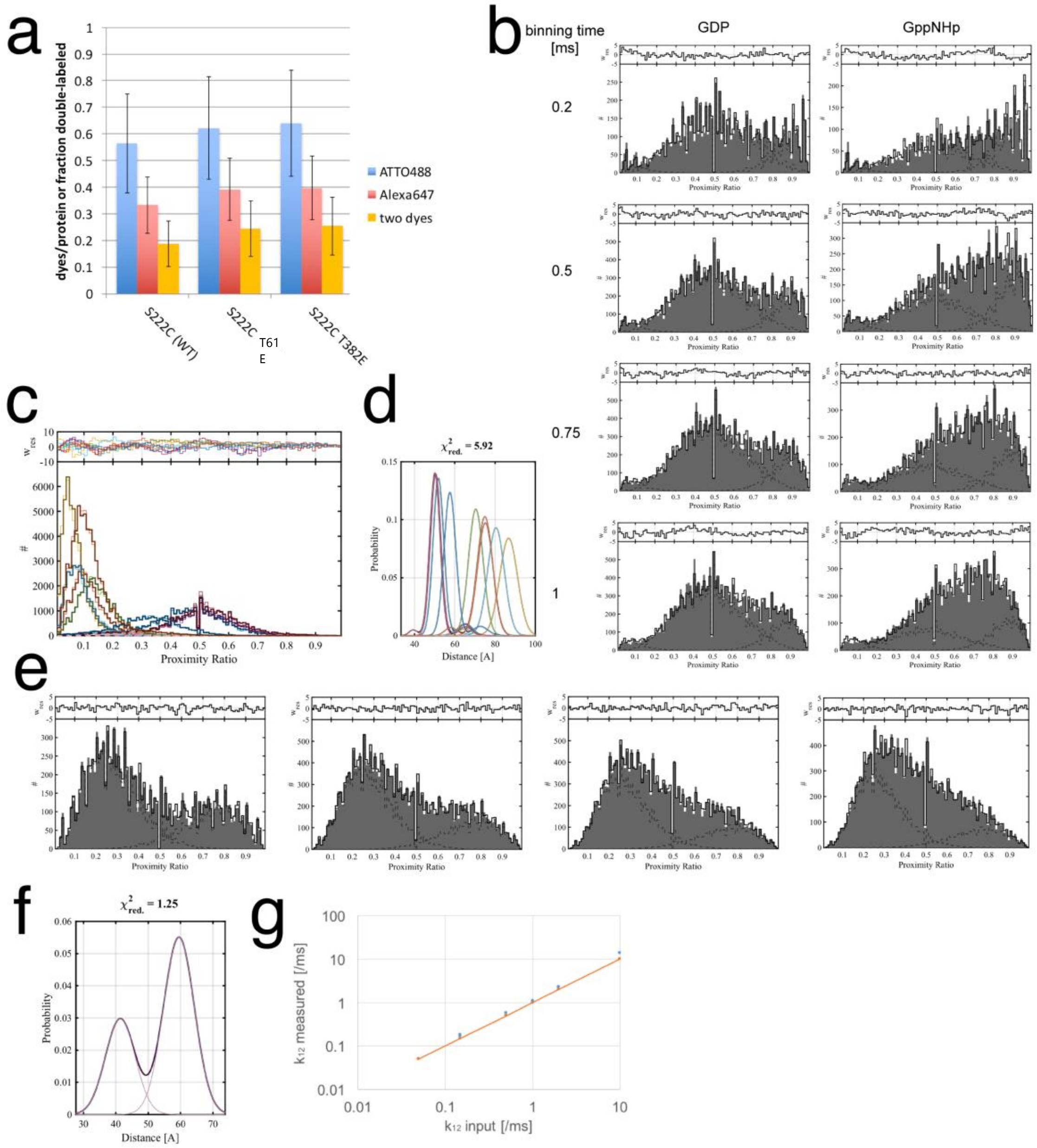

fig. S6. Characterization of the labeling of EF-Tu with ATTO 488 and Alexa Fluor 647. Typical result from a fluorescent labeling experiment of EF-Tu as measured by absorbance spectrophotometry (Biodrop, Isogen Life Science, de Meern, The Netherlands) analysis. The A_{280} was corrected for the contribution of both dyes, and concentrations were subsequently calculated using ε_{280nm} = 20,525 $(cm\cdot M)^{-1}$ for the protein, ε_{500nm} = 90,000 $(cm\cdot M)^{-1}$ for the ATTO 488 dye and ε_{650nm} = 260,000 $(cm\cdot M)^{-1}$ for the Alexa Fluor 647 dye. The n.o. dyes per protein was calculated from the ratio of dye vs. protein concentration, and an estimation of the fraction of double-labeled protein naively as the product of those two ratios. The error bars were calculated from the estimated error δA = 0.01 on the absorbance measurement. (b) Complete illustration of the data and dynamic PDA fit of WT EF-Tu. Burst data was rebinned in either of four time bins (left of the histogram). Overall χ^2 was 1.97. Correction parameters were gamma = 0.693, crosstalk = 0.007, BG_D = 1.99 kHz, BG_A = 0.85 kHz and R_0 = 53 Å. Especially for the GDPNP data, a separation of the histogram into two species is obvious when the binning time decreases. (c-d) PDA analysis on different 40-bp dsDNA

molecules labelled with the same dyes as the protein (ATTO 488 and Alexa Fluor 647) at well-defined spacing. (c) Static PDA analysis with gaussian distribution widths globally determined as a distance fraction *F* over the total range of *R* values. (d) Corresponding distance distribution plots illustrating the intricate relation of distance and FRET distribution width. Additionally, a dynamic PDA analysis of these conformationally static molecules indeed resulted in interconversion rate constants near zero (data not shown). (e-g) SpFRET simulation experiment to verify the range of interconversion rate constants that can, in principle, be determined using dynamic PDA. (e) Exemplary analysis: k_{12} = 1/ms and k_{21} = 0.5/ms, low signal-to-noise. From left to right 0.2, 0.5, 0.75 and 1-ms binning time. (f) Distance distribution plot. (g) Accuracy of k_{12} values obtained by dynamic PDA. Orange line has a slope = 1.

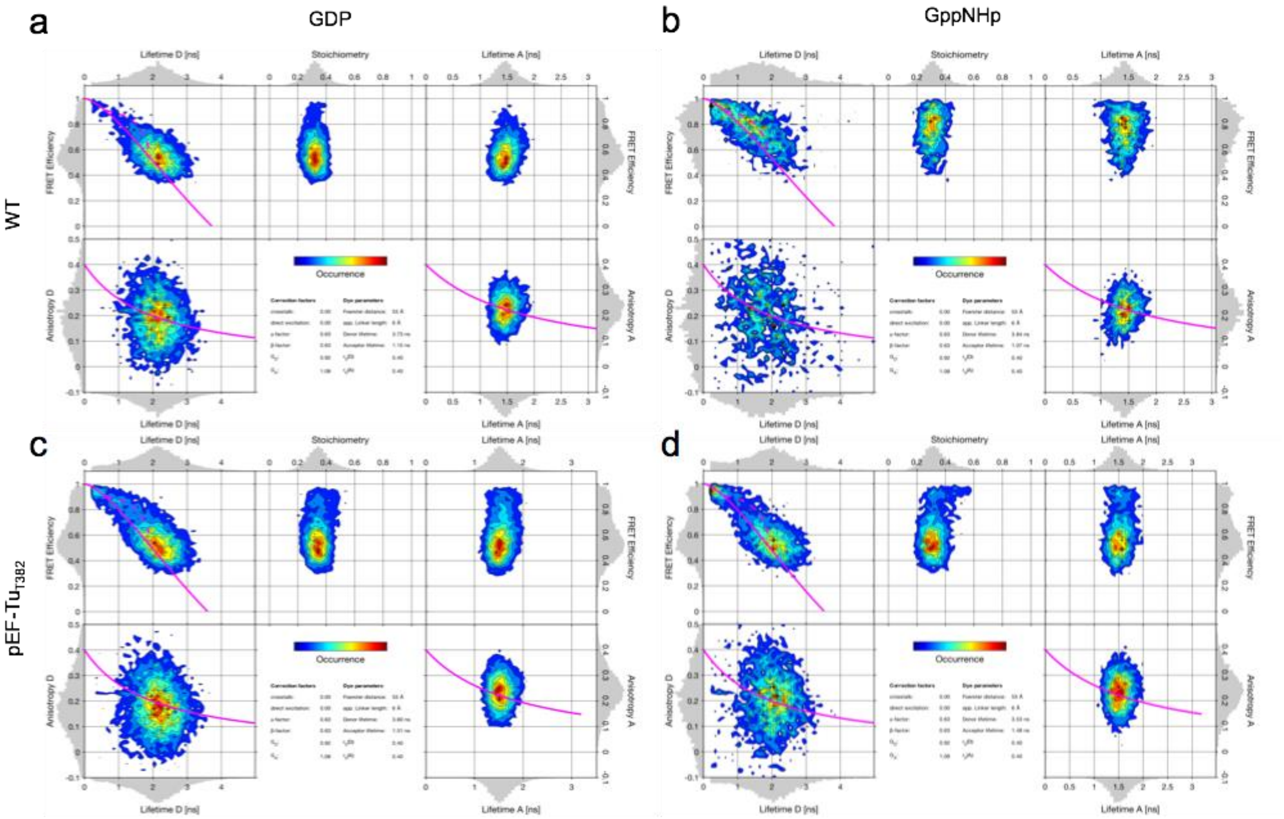

fig. S7. Multiparameter graphs obtained after spFRET analysis of EF-Tu and phosphorylated EF-Tu in the presence of GDP and GDPNP. Multiparameter graphs obtained after spFRET analysis of (**a**, **b**) EF-Tu and (**c**, **d**) pEF-Tu$_{T382}$ in the presence of GDP and GDPNP. In each panel is a multidimensional plot (2D histograms and their 1D on-axis sum) of the FRET efficiency E versus the donor fluorescence lifetime $\tau_{D(A)}$, stoichiometry S and acceptor fluorescence lifetime τ_A, a plot of the donor fluorescence lifetime versus the donor anisotropy $r_{D(A)}$, and the acceptor fluorescence lifetime versus acceptor anisotropy r_A. A 15% offset in z was used to reduce noise in the 2D histograms. Overlayed on the E *vs.* $\tau_{D(A)}$ data is (solid line) the static FRET line (calculated using a τ_D estimated from the D-only population, an R_0 = 53 Å, and assuming fast dye fluctuations over 6 Å) and (dashed line) the dynamic FRET line using two hand-picked extreme-state lifetimes. Overlayed on the τ vs. r plots is the Perrin equation plotted to go through the center of the data cloud (θ ~2 ns). All correction and calibration parameters are also indicated in the white space.

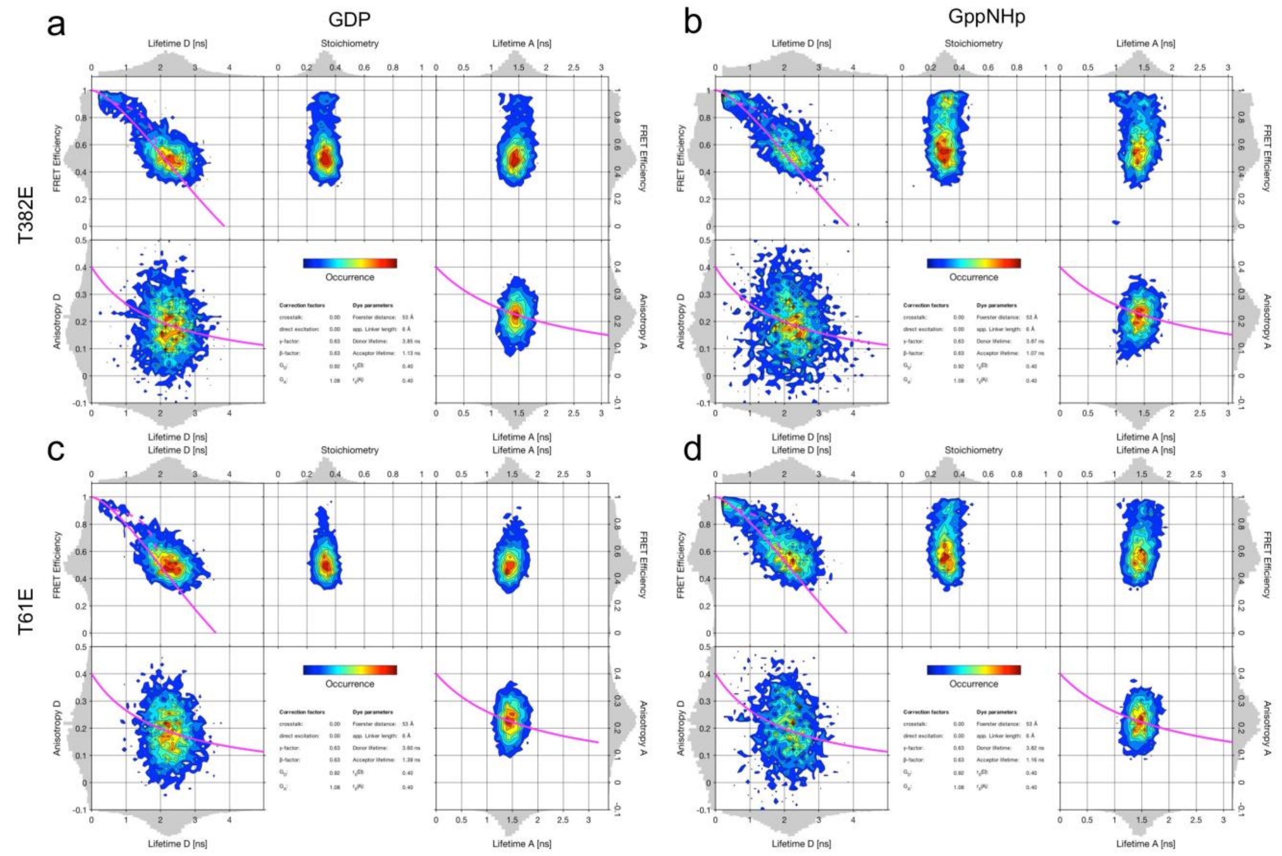

fig. S8. Multiparameter graphs obtained after spFRET analysis of the EF-Tu phosphomimetic mutants in the presence of GDP and GDPNP. Multiparameter graphs obtained after spFRET analysis of (**a**, **b**) T382E, and (**c**, **d**) T61E in the presence of GDP and GDPNP. In each panel is a multidimensional plot (2D histograms and their 1D on-axis sum) of the FRET efficiency E versus the donor fluorescence lifetime $\tau_{D(A)}$, stoichiometry S and acceptor fluorescence lifetime τ_A, a plot of the donor fluorescence lifetime versus the donor anisotropy $r_{D(A)}$, and the acceptor fluorescence lifetime versus acceptor anisotropy r_A. A 15% offset in z was used to reduce noise in the 2D histograms. Overlayed on the E *vs.* $\tau_{D(A)}$ data is (solid line) the static FRET line (calculated using a τ_D estimated from the D-only population, an R_0 = 53 Å, and assuming fast dye fluctuations over 6 Å) and (dashed line) the dynamic FRET line using two hand-picked extreme-state lifetimes. Overlayed on the τ vs. r plots is the Perrin equation plotted to go through the center of the data cloud (θ ~2 ns). All correction and calibration parameters are also indicated in the white space.

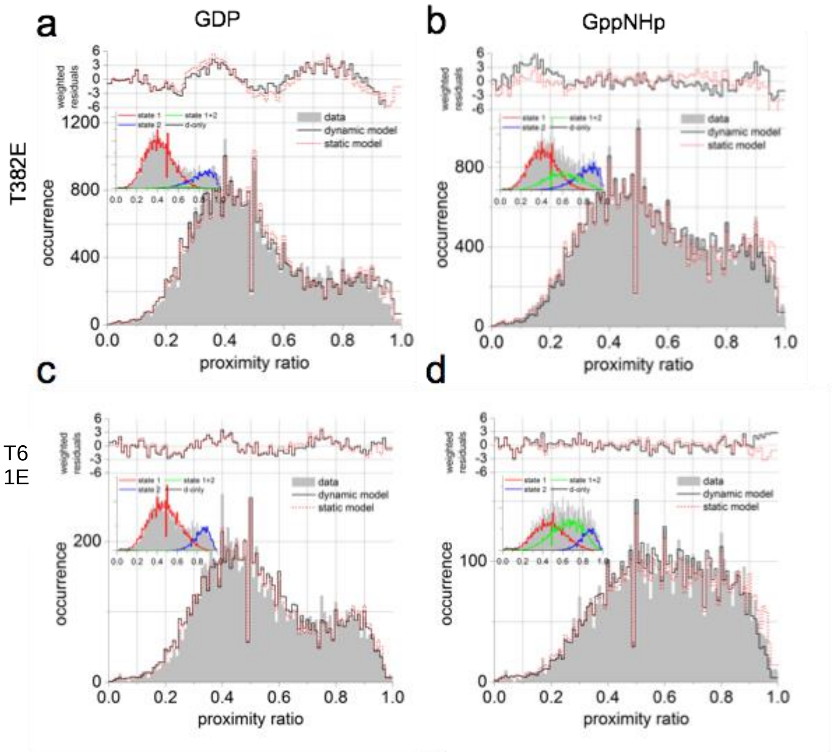

fig. S9. Static versus dynamic PDA analysis of the EF-Tu phosphomimetic mutants in the presence of GDP and GDPNP. Static vs. dynamic PDA analysis of (a,b) T382E and (c,d) T61E in the presence of GDP and GDPNP. The insets of the static versus dynamic PDA analysis illustrate the sub-states used for dynamic PDA analysis, *i.e.* the relative abundance of molecules that coincidently were only in the (red) open or (blue) closed state, and molecules that (green) interconverted from one state to the other during diffusion through the probe volume. The (relative) area under the green substrate is directly proportional to the chance of observing a molecule that interconverted while diffusing through the probe volume; the larger this area, the more robustly can kinetic rate constants be derived from the data by dynamic PDA.

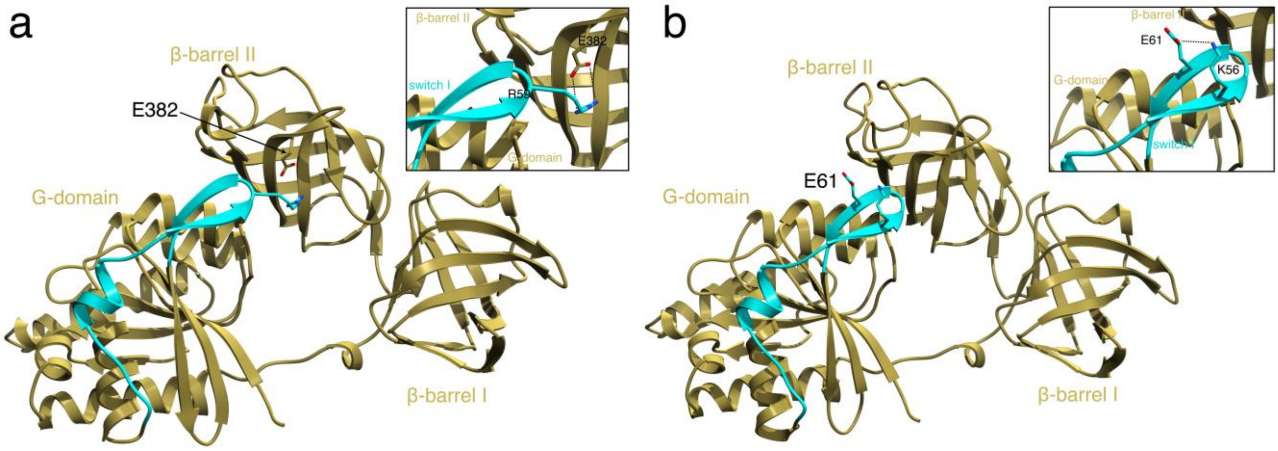

fig. S10. X-ray structure of the EF-Tu phosphomimetic mutants. EF-Tu$_{T382E}$ (**a**) and EF-Tu$_{T61E}$ (**b**) are represented in the style of Fig. 3a highlighting each individual domain of EF-Tu and showing the switch I region in cyan. In both cases the inset details the local interactions stabilizing the switch I region (E382-R59 for EF-Tu$_{T382E}$ and E61-K56 for EF-Tu$_{T61E}$).

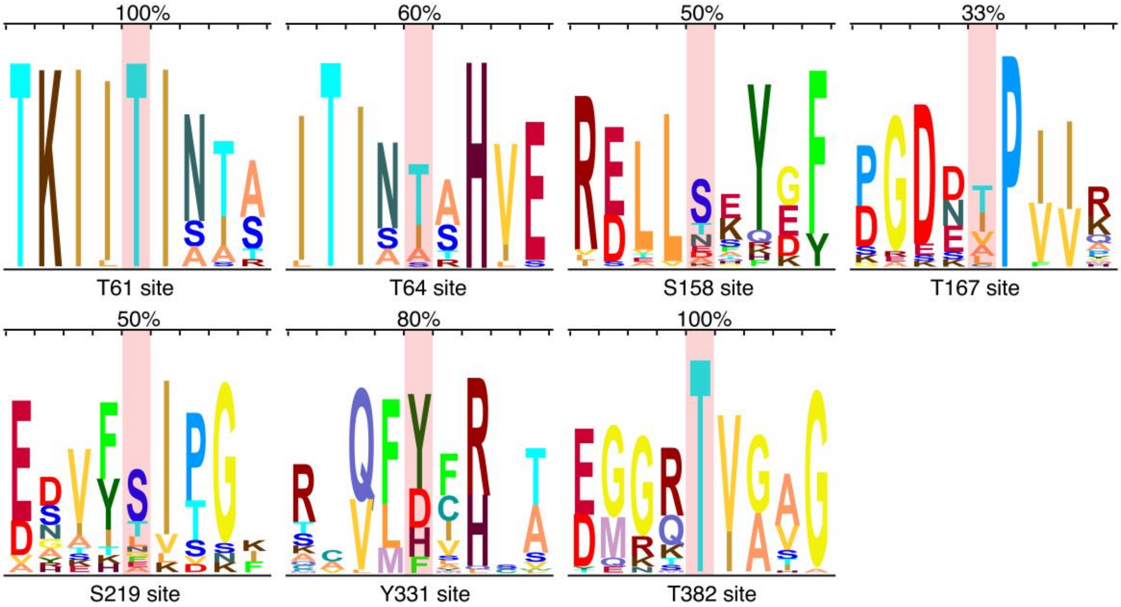

fig. S11. Analysis of the conservation of the phosphorylation sites across the EF-Tu superfamily. The degree of conservation of each site is expressed as logos that represent the sequence alignment. The logos are stacks of letters for each position, where the height of the stack corresponds to the conservation at that position, and the height of each letter within a stack depends on the frequency of that letter at that position. As shown in the figure, there are several sites (including T61 and T382) that are highly conserved across the super-family.
